# FAIR Genomes metadata schema promoting Next Generation Sequencing data reuse in Dutch healthcare and research

**DOI:** 10.1038/s41597-022-01265-x

**Published:** 2022-04-13

**Authors:** K. Joeri van der Velde, Gurnoor Singh, Rajaram Kaliyaperumal, XiaoFeng Liao, Sander de Ridder, Susanne Rebers, Hindrik H. D. Kerstens, Fernanda de Andrade, Jeroen van Reeuwijk, Fini E. De Gruyter, Saskia Hiltemann, Maarten Ligtvoet, Marjan M. Weiss, Hanneke W. M. van Deutekom, Anne M. L. Jansen, Andrew P. Stubbs, Lisenka E. L. M. Vissers, Jeroen F. J. Laros, Esther van Enckevort, Daphne Stemkens, Peter A. C. ‘t Hoen, Jeroen A. M. Beliën, Mariëlle E. van Gijn, Morris A. Swertz

**Affiliations:** 1 University of Groningen and University Medical Center Groningen, Genomics Coordination Center, Antonius Deusinglaan 1, 9713 AV Groningen, The Netherlands; 2 University of Groningen and University Medical Center Groningen, Department of Genetics, Antonius Deusinglaan 1, 9713 AV Groningen, The Netherlands; 3 Radboud University Medical Center, Radboud Institute for Molecular Life Sciences, Center for Molecular and Biomolecular Informatics, Geert Grooteplein 28, 6525 GA Nijmegen, The Netherlands; 4 Leiden University Medical Center, Department of Human Genetics, Einthovenweg 20, 2333 ZC Leiden, The Netherlands; 5 Amsterdam University Medical Center, University of Amsterdam, Department of Pathology, Meibergdreef 9, 1105 AZ Amsterdam, The Netherlands; 6 The Netherlands Cancer Institute, Division of Molecular Pathology, Plesmanlaan 121, 1066 CX Amsterdam, The Netherlands; 7 Prinses Máxima Center for Pediatric Oncology, Kemmeren group, Heidelberglaan 25, 3584 CS Utrecht, The Netherlands; 8 Radboud University Medical Center, Department of Human Genetics, Donders Institute for Brain, Cognition and Behaviour, Geert Grooteplein 10, 6525 GA Nijmegen, The Netherlands; 9 University Medical Center Utrecht, Department of Genetics, Heidelberglaan 100, 3584 CX Utrecht, The Netherlands; 10 Erasmus Medical Center, Department of Pathology, Doctor Molewaterplein 40, 3015 GD Rotterdam, The Netherlands; 11 Nictiz - Dutch competence centre for electronic exchange of health and care information, Oude Middenweg 55, 2491 AC The Hague, The Netherlands; 12 Radboud University Medical Center, Department of Human Genetics, Geert Grooteplein 10, 6525 GA Nijmegen, The Netherlands; 13 University Medical Center Utrecht, Department of Pathology, Heidelberglaan 100, 3584 CX Utrecht, The Netherlands; 14 Leiden University Medical Center, Department of Clinical Genetics, Einthovenweg 20, 2333 ZC Leiden, The Netherlands; 15 Rijksinstituut voor Volksgezondheid en Milieu, Antonie van Leeuwenhoeklaan 9, 3721 MA Bilthoven, The Netherlands; 16 VSOP - Patient Alliance for Rare and Genetic Diseases The Netherlands, Koninginnelaan 23, 3762 DA Soest, The Netherlands; 17 Amsterdam University Medical Center, Vrije Universiteit Amsterdam, Department of Pathology, De Boelelaan 1117, 1081 HV Amsterdam, The Netherlands

**Keywords:** Standards, Genetic testing, Oncogenesis, DNA, Scientific data

## Abstract

The genomes of thousands of individuals are profiled within Dutch healthcare and research each year. However, this valuable genomic data, associated clinical data and consent are captured in different ways and stored across many systems and organizations. This makes it difficult to discover rare disease patients, reuse data for personalized medicine and establish research cohorts based on specific parameters. FAIR Genomes aims to enable NGS data reuse by developing metadata standards for the data descriptions needed to FAIRify genomic data while also addressing ELSI issues. We developed a semantic schema of essential data elements harmonized with international FAIR initiatives. The FAIR Genomes schema v1.1 contains 110 elements in 9 modules. It reuses common ontologies such as NCIT, DUO and EDAM, only introducing new terms when necessary. The schema is represented by a YAML file that can be transformed into templates for data entry software (EDC) and programmatic interfaces (JSON, RDF) to ease genomic data sharing in research and healthcare. The schema, documentation and MOLGENIS reference implementation are available at https://fairgenomes.org.

## Introduction

The FAIR principles^1^ have sparked numerous initiatives to increase the Findability, Accessibility, Interoperability and Reusability of data^2–5^. In their wake, guidelines and tools have emerged to assist FAIRification in practice^6–12^, and FAIR principles are now embraced by the life sciences^13–21^ for all types of omics^22–33^, including genomics^34–40^, and medicine to enhance the diagnostic process for rare genetic diseases^41–47^, strengthening the data management and exchange that is already integral to most research, diagnostics, and translational science in between.

Dutch pathology laboratories are accustomed to making their data FAIR, as illustrated by the current PALGA databank^48^, which comprises 55 laboratories and makes more than 42 million pathology reports findable. Dutch genome diagnostic laboratories also recently embraced FAIR principles to share over 168,000 variant classifications, resulting in accelerated and improved variant interpretation^49^. These genomic variants are detected by next-generation sequencing (NGS). However, the NGS-based genomic data itself and the associated clinical patient descriptions as well as patient informed consent for research or diagnostics are usually difficult to find and reuse because they are captured in different ways and stored across the various Dutch laboratories. This fragmented environment makes it nearly impossible for healthcare users to discover particular patients or reuse data for personalized medicine purposes such as drug response or disease risk prediction. Furthermore, in a research setting, establishing cohorts based on specific parameters is time-consuming for the same reasons. In the first quarter of 2018, sparked by an analysis from Dutch funding agency ZonMw, the ZonMw GGG Personalised Medicine research program (https://www.zonmw.nl) asked for a gap analysis to be performed to identify current standards and obstacles in optimal NGS data management according to FAIR principles^50^. To overcome these obstacles and improve healthcare benefits and research progress, 14 Dutch medical centers and institutes have now joined forces in the Dutch FAIR Genomes project.

After an initial gap analysis, FAIR Genomes set out to reach consensus on aspects important for optimal (re)use of NGS data for the research and healthcare institutes in the Netherlands that perform NGS to uncover germline or somatic DNA variation. With a wide group of stakeholders, ranging from clinical geneticists and basic researchers to laboratory directors and patient organizations, we developed data and metadata standards to improve genomic data findability and allow for accessing sensitive data while protecting privacy. Here, we present the FAIR Genomes metadata schema and prototypes of data capture systems based on this schema as a foundation to promote NGS FAIRification in the Netherlands. This work was based on a comparison of local genomic data flows against a shared reference architecture across 66 Dutch stakeholders (see Supplementary Data S1). The (meta)data standards will encompass crucial information such as technical, clinical and biological specifications, conditions under which data repurposing is allowed, and facts about the involved people or patients, so that it is immediatly clear if data may be reused to support a particular research project or diagnostic question. Upon accessing the underlying standardized data, laborous tasks may be automated, including genetic or phenotypic matchmaking to solve rare disease cases or the processing and analysis of relevant samples to discover new knowledge for clinical benefit.

## Results

The approach used to create the FAIR Genomes metadata schema can be summarized in four steps: (i) we reached consensus among all participating centers about which metadata elements are important for finding and reusing NGS data, (ii) we built the semantic schema to capture these elements in both human-readable and machine-readable ways, (iii) we developed solutions and resources to translate the schema into practical and interoperable systems in a heterogeneous IT landscape and (iv) we created prototype FAIR Genomes‒compatible systems that were tested and refined in pilot projects and we then started FAIRification in practice.

### Reaching consensus and building the semantic schema for NGS harmonization

**Fig. 1:**
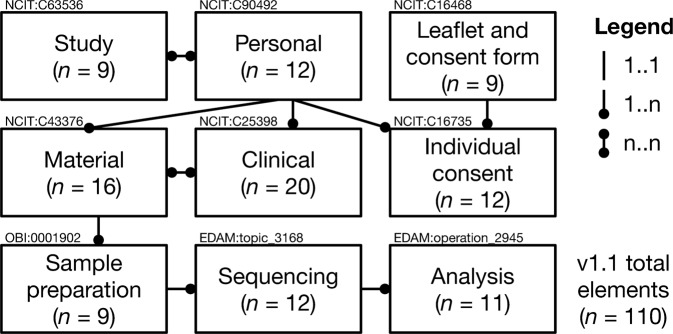
Overview of FAIR Genomes v1.1 modules, including their cardinality (i.e. the links between the modules) and semantics (i.e. ontological annotations). This schema follows the typical flow of an NGS analysis in molecular diagnostics or research.

After several inventory and consensus meetings with 66 different stakeholders—including clinicians, data stewards, researchers, laboratory specialists, bioinformaticians and patient organizations—the FAIR Genomes Consortium (members listed in Supplementary Data S1) reached a consensus on which data elements are vital for discovering, sharing and reusing NGS data. This resulted in 110 data elements that were grouped into 9 modules: *Study*, *Personal*, *Leaflet and consent form*, *Individual consent*, *Clinical*, *Material*, *Sample preparation*, *Sequencing* and *Analysis*. Figure 1 shows an overview of the number of elements per module and how they are linked. Supplementary Data S2 provides a specification of the FAIR Genomes schema v1.1.

The 110 data elements have preferred value types such as integer, string or date. There is also a special element value type called ‘lookup’ that refers to a set of all user-selectable options. All modules, elements and lookups are defined using ontology terms to prevent ambiguity in the meaning of the concepts used. In addition, these definitions allow computer-readable formats to be created. Also, using the ‘reference’ value type, modules may refer to other modules as their source, in essence following the typical flow of an NGS analysis in diagnostics or research. For instance, *Sample preparation* is performed on a *Material* taken from a *Person*.

Not all terms needed to build the FAIR Genomes schema were present in existing ontologies. Therefore, to complete the schema and lookups, we defined 743 new ontology terms. Of these terms, 740 are new lookup values for data-use modifiers, institutes, NGS kits, tissue pathological state, and sequencing instrument models, while three terms represent new element definitions: *Intended Insert Size* and *Observed insert size* in the *Sequencing* module, and *Percentage TR20* in the *Analysis* module.

Documentation for the FAIR Genomes schema can be accessed in different formats. The HTML rendered from Markdown format is a human-friendly interactive overview of the schema and all of its modules and elements. It includes summary statistics and hyperlinks to ontology references and lookup lists. The LaTeX format offers a simplified module overview and is accompanied by a script to produce a typeset PDF document that is suitable for printing and publishing.

**Fig. 2:**
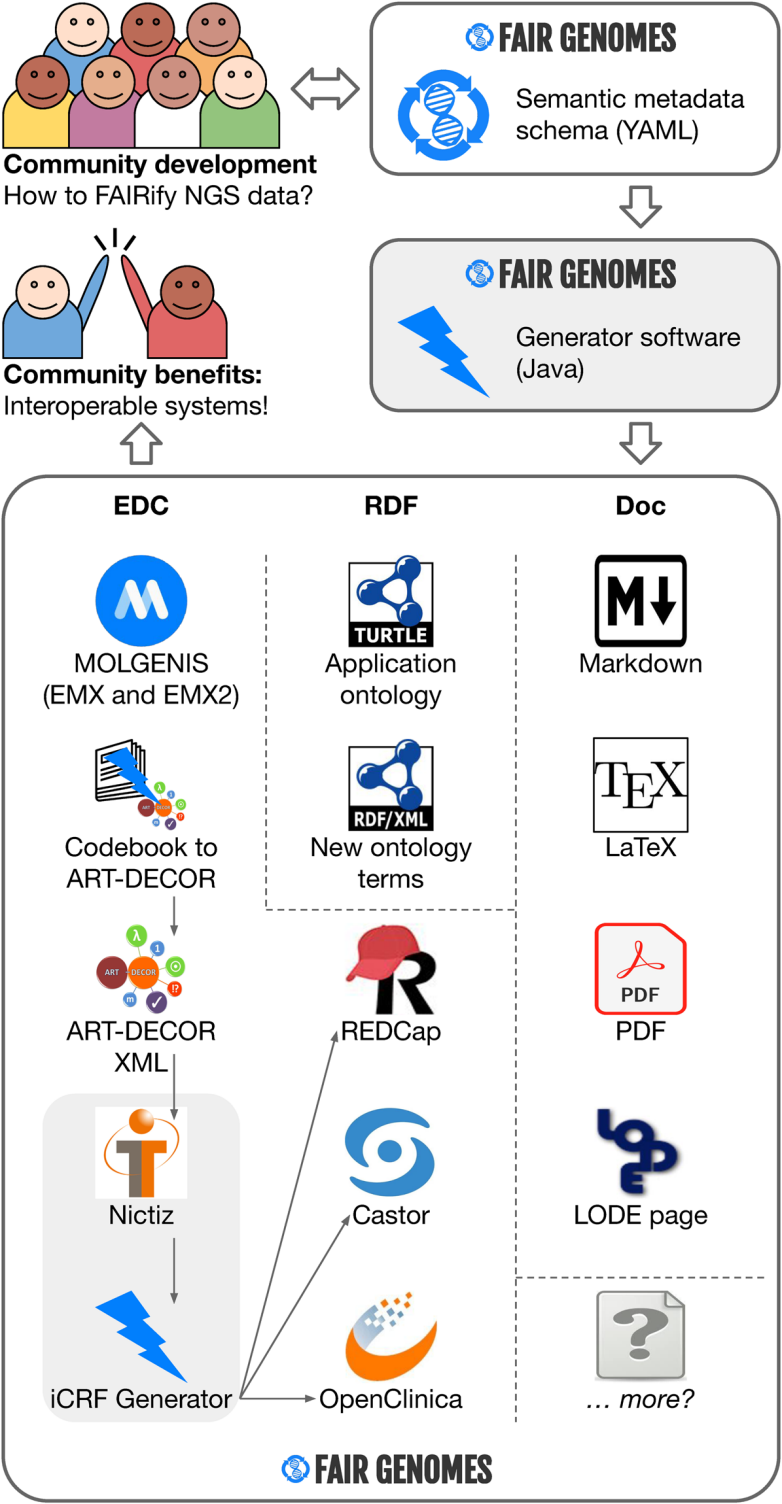
The flow from FAIR Genomes schema development to interoperable systems. The community can focus their efforts on defining a metadata schema. The resulting schema then feeds into a software generator that takes care of the required EDC blueprints, semantic resources and documentation. As a result, the community benefits from systems that are directly interoperable and ‘FAIR at the source’.

The FAIR Genomes schema is intended as an evolving standard. Both the schema and lookups are presented as versioned releases to ensure stability while being improved through an open-source repository and issue tracker at https://github.com/fairgenomes/fairgenomes-semantic-model. The schema is stored as a YAML file (https://yaml.org) that serves as input for an automated software generator. Running the generator produces resources for Electronic Data Capture systems (EDCs) (for humans and computers), an application ontology (for computers) and accompanying documentation as a basis for prototypes (see Fig. 2). Also a reference implementation in MOLGENIS is maintained, which is also used for demonstration purposes. These are discussed in more detail below.

### Blueprints for data-capture systems

Many EDCs allow part of their data model to be defined at runtime because data capture needs are often too diverse to be supported by a fixed predefined model. These runtime definitions are typically accomplished by importing a data model template (i.e. blueprint) into the EDC. After model validation and configuration (depending on the system), the EDC is ready to receive data that conforms to this model. Data input may then take place using programmatic or human interfaces such as data entry forms that are all based on the same blueprint.

We have developed software to parse the FAIR Genomes schema and generate blueprints to set up EDCs for recording FAIR Genomes data ‘at the source’. We currently offer direct support for the EDCs most widely used for research consented reuse of patient data within Dutch academic hospitals, including MOLGENIS^51,52^, Castor^53^, REDCap^54,55^ and OpenClinica3^56^. In addition, FAIR Genomes may be implemented in HL7/FHIR-based standards using the ART-DECOR® framework^57^. By using FAIR Genomes blueprints, data capture systems become more interoperable and cross-queryable, reducing or even removing the need to perform data extraction, transformation and loading into a centralized database in order to find and reuse data. Various FAIR Genomes EDC prototypes are listed below.

MOLGENIS is an EDC and web-based data platform used in many life science applications, including biobank catalogues, patient registries, diagnostic tools and research databases. A local ready-to-use MOLGENIS FAIR Genomes database can be created using an automated installation script. A Docker image is available that fully automates this process (see Supplementary Data S3). Supplementary Data S4 provides a walkthrough of the FAIR Genomes MOLGENIS EDC. The next generation of MOLGENIS software is currently in development and available as a beta version at https://github.com/molgenis/molgenis-emx2. It uses a more compact and semantically rich (meta)data format (MOLGENIS-EMX2) as compared to the format used by the previous generation of MOLGENIS software (MOLGENIS-EMX). FAIR Genomes is available as a MOLGENIS-EMX2 format in anticipation of future use.

Castor, REDCap, and OpenClinica3 are clinical EDCs supported by FAIR Genomes. Forms for these EDCs can be composed by iCRF Generator^58^ via a step-by-step interactive wizard. The iCRF Generator provides access to several well-known codebooks stored in ART-DECOR® and allows users to select items from these codebooks to build their own case report forms. Supplementary Data S5 contains a description of how to install and use iCRF Generator to generate EDC forms using the FAIR Genomes schema. We have also created step-by-step walkthroughs of how to import forms created by iCRF Generator into EDCs for Castor (Supplementary Data S6) and REDCap (Supplementary Data S7).

To enable iCRF Generator to build EDC forms, the FAIR Genomes schema has been uploaded into an ART-DECOR® platform. ART-DECOR® is an open-source tool suite that supports the creation and maintenance of HL7/FHIR templates, value sets, scenarios and data sets. The FAIR Genomes is hosted as a ‘Dataset’ by Nictiz, a Dutch knowledge organization for digital information exchange in healthcare (https://www.nictiz.nl) at https://decor.nictiz.nl/art-decor/decor-datasets--fairgenomes-. The ART-DECOR® resource can be reused in other contexts for achieving interoperability in healthcare because it is a structured standard. The ART-DECOR® resource can also be used in HL7/FHIR integration efforts, which is the international standard for data transfer between Electronic Health Record (EHR) systems.

### Application ontology

The complete FAIR Genomes schema and all of its lookups are available as an application ontology, which represents a consistent human-readable and machine-readable knowledge base of FAIR Genomes modules, concepts, and their definitions and associated properties like domain and range. The application ontology is expressed in Turtle (TTL) format, a highly efficient way of writing down data in the Resource Description Framework (RDF, https://www.w3.org/RDF). RDF captures information in a computer-readable way as subject-predicate-object nodes known as triples. Triples can be connected to other triples to form graphs from which new knowledge may be inferred. In FAIR Genomes v1.1, the application ontology is divided into a core schema file containing the definitions of modules and elements that is supported by 28 files for each lookup list.

The FAIR Genomes ontology imports concepts from existing ontologies such as NCIT, DUO, and EDAM, without alternating their meaning. New ontology terms, such as data usage modifiers, institutes, and sequencing instrument models, were introduced to complete the schema. These new terms are formally defined by RDF-XML fragments, each containing exactly one term. They are referenced in the schema and lookups by declaring Internationalized Resource Identifiers (IRIs) that are redirected via a W3ID namespace to provide URL stability. For instance, https://w3id.org/fair-genomes/resource/FG_0000001 will resolve in a fragment that describes *Intended insert size*. The application ontology and term definitions can be used directly in triple-store databases such as GraphDB. When accessed via a web browser, RDF-XML fragments are transformed into human-readable HTML pages by an SIO-based^59^ XSLT stylesheet.

A Live OWL Documentation Environment^60^ (LODE) page was created from the application ontology. LODE renders RDF vocabularies into a human-readable HTML page with ordered lists of classes, properties, annotations, and so on. It offers technical readers a familiar and predictable way to browse ontologies. The FAIR Genomes LODE page is built from the core schema, with the supporting lookup lists included via hyperlinks. Furthermore, the FAIR Genomes application ontology was published on BioPortal (https://bioportal.bioontology.org/ontologies/FG) and on FAIRSharing (https://fairsharing.org/bsg-s001533/).

### Prototype systems

To evaluate and refine the schema, we developed a number of prototype demonstrators and pilot systems. In addition, we actively exchanged modeling decisions with the designers of multiple existing systems to ensure interoperability with ‘best practice’ examples, including the RD3 database in collaboration with the SolveRD project, the Trecode database at Prinses Máxima Center for Child Oncology, and the COSAS database at University Medical Center Groningen.

A FAIR Genomes blueprint was created for the MOLGENIS database framework^51,52^. Using this blueprint, a demonstrator was created by running the default installation procedure on a MOLGENIS 8.6.2 server, resulting in an application with open permissions that allows any interested individual to access and evaluate the database at https://fairgenomes-acc.gcc.rug.nl. In addition, a production system based on FAIR Genomes v1.0 was deployed at https://fairgenomes.molgeniscloud.org. This system is currently used to collect sample metadata across all FAIR Genomes modules from the Dutch Rare Disease Consortium RADICON-NL WGS-first project.

The RD3 database for SolveRD was developed in parallel with the FAIR Genomes project. It contains rich metadata on NGS subjects, samples, lab processes and files from the SolveRD project^61^, which performs re-analysis of >19,000 unsolved rare disease patients. Data freeze 1 of SolveRD includes data from 8,393 individuals^62^. The data structure had been kept compatible with FAIR Genomes for elements concerning phenotypes, absent phenotypes, recontact details, family and sex information, tissue and material types, anatomical locations, file types and locations, enrichment kits, type of sequencing, studies, and so on. The database is located at https://solve-rd.gcc.rug.nl/.

The Trecode database^36^ was developed at the Princess Máxima Center for Pediatric Oncology following the FAIR Guiding Principles^1^. This project and FAIR Genomes influenced each other’s modeling decisions. Trecode features a bespoke data structure that includes many elements shared with FAIR Genomes, such as individual, biomaterial, library and analysis. Notable differences include more detailed computational pipelines and file tracking that are currently not within the scope of FAIR Genomes. The Trecode programs, data models and workflows are in the process of being made open and reusable in the public domain.

The Catalogue of Sequencing and Array Samples (COSAS) database is currently in development at the Dept. of Genetics of the University Medical Center Groningen. Even though data sharing and reuse are not primary goals of local hospital administration systems such as COSAS, applying the FAIR principes is still necessary to ensure local findability, accessibility, and interoperability. Therefore, the COSAS model is based on FAIR Genomes and contains classes for patients, samples, lab information and files, with attributes such as date of birth, self-reported sex and biological sex, material type, phenotype, consanguinity, disease codes, sequencing type, the capture and preparation kit used and file types stored. COSAS has an explicit mapping to FAIR Genomes to maintain close integration.

Finally, various sets of NGS metadata are currently being expressed in FAIR Genomes-compliant representations, including data from the national primary immunodeficiency (PID) study, the influencing progression of airway disease in patients with primary antibody deficiency study (IPAD trial), VKGN/VKGL Dutch national diagnostic consent working group, and whole-exome and whole-genome sequencing samples from several collaborating institutes, stored locally using local frameworks.

Links and information for all resources and prototypes can be accessed via the FAIR Genomes website at https://fairgenomes.org.

## Discussion

We developed the FAIR Genomes metadata schema based on a national consensus amongst stakeholders in the Netherlands on the data elements necessary to facilitate sharing of NGS data. Where possible we learned from international initiatives such as GA4GH, European Joint Programme for rare disease (EJP-RD) and existing large public databases, adapting existing elements to the FAIR Genomes schema. This semantic schema can be used to generate blueprints for a variety of data-capture systems and makes data exchange interoperable. Using prototypes, we have now demonstrated that this approach is feasible. Therefore, although the schema and technology will continue to be developed in collaboration with (inter)national partners, we conclude that we have laid a solid foundation to start implementing FAIR Genomes-compatible production systems for FAIRification in practice. In addition, by involving stakeholders, we increased awareness of the FAIR principles amongst healthcare professionals, and we expect that our efforts will contribute to considerably more reuse of NGS data between Dutch institutes and provide a firm basis for semantic interoperability of European genome data, e.g. as a promising best practice within the 1 + Million Genomes (1 + MG) and Beyond 1 Million Genomes (B1MG) initiatives^63^.

However, FAIRification is not a trivial process, and challenges will arise when adopting schemas such as FAIR Genomes, especially considering the heterogeneity of the IT landscape surrounding NGS data. A primary concern is how to make existing data systems FAIR Genomes-compliant for both retrospective and prospective use without data conversion or recoding efforts. This challenge can be overcome by using the semantics (i.e. ontology terms) of FAIR Genomes to ‘tag’ tables (modules), attributes (elements) and rows (lookups) within an existing system without modifying the source data. In this way, interoperability with similarly tagged data could be introduced. For instance, in MOLGENIS-EMX2, the ‘semantics’ property can associate database records to ontologies, enabling RDF exports that are just rich enough for interoperability. Moreover, in the Castor EDC, existing studies can be mapped to semantic schemas for FAIRification^44^. If the source database does not offer such capabilities, RML templates^64^ offer a generic solution that allows CSV to be transformed into RDF. When multiple EDCs or EHRs adhere to the same schema, interoperability amongst these systems will be high. However, without a harmonized Application Programming Interface (API), federated queries may still require manual data extraction and loading. FAIR Data Point^65^ technology alleviates this problem by enabling automated discovery and standardized access to the data. MOLGENIS-EMX, MOLGENIS-EMX2 and Castor support setting up a FAIR Data Point (see Methods). To our knowledge, REDCap and OpenClinica do not support FAIR Data Point yet. Further interoperability enhancements that would allow FAIR Genomes to better relate to EHRs and other health applications may include alignment with Health and Care Information models (HCIM, https://zibs.nl) and openEHR archetypes^66^.

Another challenge is how to deal with future updates of the FAIR Genomes schema that may be partially incompatible with older versions. This problem will be mitigated by mapping existing terms to additional ontologies using the SKOS vocabulary^67^ (e.g. *exactMatch*, *closeMatch*, *relatedMatch*) instead of replacing these mappings, and deprecating terms instead of deleting terms that should no longer be used. In practice, we expect that any non-backwards compatible updates will only affect a small fraction of the schema, causing a minimal loss of interoperability that can be easily restored, especially in the case of data tagging. Even systems that only reach partial FAIR Genomes compatibility can still benefit from a shallower form of tagging. For example, tags can be used on element-level while different code systems are used underneath (e.g. using FMA instead of UBERON to capture an *Anatomical source*). Queries and retrieval across these systems will then remain possible, albeit with some limitations.

Temporality is an important aspect of capturing data using schemas such as FAIR Genomes. Certain values may change over time, e.g. patient phenotypes may appear or disappear, consent may be given or withdrawn, or medication may change, and even seemingly static data such as country of birth may be temporal if data entry mistakes are later corrected. We chose to not include temporality as a rule, because this may depend on implementation-specific requirements. Moreover, each element may be temporal for auditing purposes, i.e. when a full data modification history is stored for traceability. The one exception to this rule is *Individual consent*, which comes with *Valid from* and *Valid until*, because this information is critical for determining if and how data may be reused.

Development of the FAIR Genomes schema has taken place in the context of human genomics. This has resulted in a human-centered schema, but we are eager to expand into other organisms to achieve cross-species interoperability. We envision future adjustments to the schema that will enable FAIR data collection for any organism. These adjustments may include the extension of smaller lookup lists (e.g. adding non-human reference genomes and ancestries), providing alternatives for larger lookup lists (e.g. organism-specific genes and phenotypes), generalization of terms (e.g. renaming *Personal* to *Subject*), redefinition of terms (e.g. broaden *Functioning* beyond human patients), and re-evaluation of terms originally intended for humans that are actually applicable to most diploid organisms (e.g. family members, gender and sex). Previous efforts have shown the usability of cross-species data-reporting standards to annotate genome sequences, genome annotations, gene descriptions, biological samples and sequences^68^.

Finally, the use of IRIs for unambiguous identification of concepts is not always straightforward. We opted for terms with semantically resolvable IRIs, i.e. those with hyperlinks pointing directly at a structured definition of this specific term. For instance, we replaced non-resolvable MIABIS-2.0-22 lookup values for *Inclusion Criteria* with resolvable OBI terms. However, terms that resolve to non-specific locations are occasionally the best fit, for instance the SPRECv3.0 codes for storage conditions. In practice, concept identification is a balance between term appropriateness and semantic resolvability. A more fundamental problem with concept identifiers is hosting. We now use a W3ID redirect on a stable namespace, but the existence of the terms and their definitions are hosted in repositories maintained by the authors of this manuscript, and the permanence of this hosting situation is not strictly guaranteed by anyone. These concepts may therefore not be persistent, which might be problematic. We can only build a knowledge network when its foundations are truly persistent. A last point concerning IRIs is that we must also uniquely identify actual persons, samples and measurements in addition to the concepts used to describe them. For instance, the same sample may appear in two FAIR databases. Without a way to link these records, valuable associations may be lost. To resolve this issue, we suggest that FAIRification projects should agree on adopting (inter)national identifiers such as BioSample^69^ for samples, EGA^70^ for files or European Digital Identity^71^/EUPID^72^ for patients. When handling and linking patient data however, the changes and increasing international differences in data privacy legislation present another major challenge, in additional to the technical challenge of privacy-preserving record linkage^73,74^. Generalized digital FAIR consent and interoperability between pseudonymisation systems are essential, but remain a complicated topic that will continue to develop and is being addressed in collaboration with the (inter)national genomics ELSI community.

The work presented here is the foundation for further FAIRification of NGS data in the Netherlands. We propose a synergistic two-pronged approach where institutes start to become ‘FAIR at the source’ in EDCs and EHRs, while at the same time the national BBMRI omics explorer (https://omics-explorer.bbmri.nl) will be renewed to showcase non-sensitive information about NGS samples that has been made nationally discoverable by FAIR Genomes. Feedback and contributions to FAIR Genomes are highly appreciated, including the creation of issues or pull requests on the open source GitHub repository at https://github.com/fairgenomes. The FAIR Genomes schema will continue to develop, and we expect to expand into FAIRification of computational pipelines and other omics (e.g. transcriptomics, proteomics, metabolomics and microbiomics) in frameworks such as X-omics, BBMRI, Health-RI, ELIXIR, SolveRD and the European Joint Programme for Rare Diseases. Please join us at: https://fairgenomes.org.

## Methods

### Reaching consensus on elements

The FAIR Genomes project kick-off meeting has held on February 27, 2019. Since then, consortium members and working groups from 14 different institutes have participated in a variety of recurring meetings, topic workshops, video conferences and one-on-one sessions, often together with (inter)national partners.

During the first year, construction of the consensus schema was initiated by creating a joint online Google spreadsheet to collect variables from consortium members via brainstorm sessions and workshops. The results of the gap analysis on optimal data management of NGS data according to the FAIR principles were used as input for these workshops, which included topics such as data generation, data quality, (meta)data standards, data storage, data archiving, data integration and data exchange. The criteria for selecting variables covered by FAIR principles were variables primarily used to find data (e.g. patient phenotype, sampled tissue), variables describing which resources are accessible for reuse (e.g. files, materials), variables capturing interoperability (e.g. library preparation, algorithms) and variables used to determine if the data is reusable in a particular context (e.g. informed consent for research or diagnostics, quality metrics). The *Leaflet and consent form* and *Individual consent* modules were modeled by a separate expert working group (see Author contributions).

During the second year, the list was curated and moved to a Markdown table in GitHub. Each element of the schema was linked to a GitHub issue where this element was discussed, and GitHub issues could be opened or closed depending on the outcome of the discussions. Open issues could be viewed in the GitHub issue list to keep track of overall progress. During this time, the schema was also segmented into smaller modules such as *Clinical* and *Personal* to allow focused work on specific modules and facilitate modular reuse via iCRF Generator and relational databases.

At that point, the elements in the schema were divided into ‘optional’ and ‘required’ elements. This distinction was later dropped in order to (i) not discourage users by imposing strict requirements that may not be attainable and (ii) to encourage users to share whatever information they are able, whether optional or required. To prevent users from having to leave values empty for data they cannot provide, HL7/FHIR ‘Null Flavors’ (https://www.hl7.org/fhir/v3/NullFlavor/cs.html) were introduced to the schema. Null flavors can be used to indicate precisely why a particular value could not be entered into the system, providing substantially more insight than simply leaving a field empty and allowing for a level of semantic richness even for missing data.

All of the modules, elements and lookup lists were annotated with ontologies. The ontology terms were selected via an iterative process. First, any matching ontology term was selected as a starting point. Where possible, we then replaced terms in the schema that originated from rarely used ontologies with equivalent or better fitting terms from ontologies more often used in the schema in order to reduce heterogeneity and the number of dependencies.

During the third year, the schema was moved from Markdown to a structured YAML file. Since element-level issues were mostly resolved, we moved to global issue tracking. FAIR Genomes version 1.1 was released July 20, 2021.

#### Interactions with (inter)national FAIR projects

The FAIR Genomes metadata schema was developed in collaboration with (inter)national partners and projects. At the start of the project, extensive searches were performed to discover the latest developments in FAIRification of NGS data. Relevant initiatives, if not involved already, were either contacted to initiate interactions for mutual benefit and synergy, or their work was taken into account in developing the FAIR Genomes schema. Nationally, such initiatives included FAIR Data Point, Trecode and X-omics. Internationally, examples included EJP-RD, CDE (by JRC), RD3 (for SolveRD), FAIRplus, EGA, ISA-TAB, TCGA, 1 + MG, B1MG, ICGC, CINECA, EuroGentest and GA4GH Beacon/Phenopackets. Topics for discussion included existing standards, data elements, schema harmonization and compatibility, choice of ontologies and semantic technologies, data exchange protocols, ethical/legal issues, future collaborations and extension of the FAIR Genomes schema. Further harmonization work will take place with CDE (by JRC), DCDE (by EJP-RD, ERICA, JRC), FDP and X-omics. See Table 1 for a glossary of all terms, abbreviations and acronyms.

**Table 1:** Glossary of terms, abbreviations and acronyms.

Term	Definition	Website
1 + MG	European ‘1 + Million Genomes’ Initiative	https://digital-strategy.ec.europa.eu/en/policies/1-million-genomes
ART-DECOR®	Advanced Requirement Tooling Data Elements, Codes, OIDs and Rules	https://art-decor.org
B1MG	Beyond 1 Million Genomes project	https://b1mg-project.eu
BBMRI	Biobanking and Biomolecular Resources Research Infrastructure	https://www.bbmri.nl
CDE	Common Data Elements	https://eu-rd-platform.jrc.ec.europa.eu/set-of-common-data-elements_en
CINECA	Common Infrastructure for National Cohorts in Europe, Canada, and Africa	https://www.cineca-project.eu
DCDE	Domain specific Common Data Elements	https://erica-rd.eu/event/domain-specific-common-data-elements-dcdes-curation
EGA	European Genome-phenome Archive	https://ega-archive.org
EJP-RD	European Joint Programme for Rare Disease	https://www.ejprarediseases.org
ELIXIR	European life-sciences Infrastructure for biological Information	https://elixir-europe.org
ERICA	European Rare Disease Research Coordination and Support Action consortium	https://erica-rd.eu
EuroGentest	EuroGentest: harmonizing genetic testing across Europe	http://www.eurogentest.org
FAIRplus	FAIRplus project	https://fairplus-project.eu
FDP	FAIR Data Point	https://www.fairdatapoint.org
FHIR	Fast Healthcare Interoperability Resources	https://www.hl7.org/fhir
GA4GH	Global Alliance for Genomics and Health	https://www.ga4gh.org
Health-RI	Health Research Infrastructure	https://www.health-ri.nl
HL7	Health Level Seven International	https://hl7.org
ICGC	International Cancer Genome Consortium	https://dcc.icgc.org
ISA-TAB	Investigation Study Assay (ISA) tab-delimited (TAB) format	https://isa-tools.org
JRC	Joint Research Centre	https://ec.europa.eu/jrc
MIABIS	Minimum Information About BIobank data Sharing	https://github.com/BBMRI-ERIC/miabis
RD3	Rare Disease Data about Data	https://solve-rd.molgeniscloud.org
SolveRD	Solving the unsolved Rare Diseases	https://solve-rd.eu
TCGA	The Cancer Genome Atlas	http://cancergenome.nih.gov
VKGL	Vereniging Klinisch Genetische Laboratoriumdiagnostiek	https://www.vkgl.nl
VKGN	Vereniging Klinische Genetica Nederland	https://www.vkgn.org
X-omics	The Netherlands X-omics Initiative (X-omics, pronounce as CROSS-omics)	https://www.x-omics.nl

### Building the semantic schema

The final semantic schema has been defined as a YAML file that is easy to read and edit, even for non-technical individuals, while also allowing for the nested objects and list structures needed to express a metadata schema. The schema consists of three levels of information. The first is the root level that describes the schema itself and includes information such as name, description, version, date, authors, copyright and license. The second is the module level that describes the logical schema partitions such as *Personal*, *Clinical* and *Material*. This level can be compared to the tables in a database or to classes in Unified Modeling Language (UML). Each module has a name, description, ontology reference and list of elements. The third level is the element level that contains the actual data elements. This level can be compared to table or class attributes. Each element has a name, description, ontology reference, and value type. Value types include standard data types such as String, Date, Boolean, Integer, etc. The value types ReferenceOne and ReferenceMany represent the cardinality of the modules by referring to either one or multiple instances of a module, i.e. a row in a database containing an actual person as defined by *Personal*. In relational database systems, the modules translates into tables and cardinality translates into foreign keys. Lastly, the LookupOne and LookupMany value types point to a predefined list of terms from which users may choose one or multiple options.

The lookup lists are simple TSV files (tab-separated values) with the following columns: value, description, codesystem, code, and IRI. For semantic correctness, lookup elements have an additional ‘ofType’ property that allows element definition (e.g. *Medication*, defined as NCIT:C459, “Medication”) to be separated from its lookup value type (e.g. for *Medication*, users can choose from a list of ATC codes that are collectively typed as EDAM:data_3103, “ATC code”). In FAIR Genomes v1.1, there are 35 elements with lookup values, referring to 29 unique lookup lists. The sizes of the lookup lists range from 3 (for *RepresentedBy*) to 19,203 (for *Genes*). In total, there are 85,307 lookups options across all the elements in FAIR Genomes release v1.1. Lookup lists may be reused, e.g. HPO terms are used in both *Phenotype* and *Unobserved phenotype*. There are 67,990 unique lookup values, i.e. when counting reused lists only once.

Lookups can be supplemented with null flavors from HL7/FHIR. These are 16 null flavors available in v1.1: *NoInformation*, *Invalid*, *Derived*, *Other*, *Negative infinity*, *Positive infinity*, *Un-encoded*, *Masked*, *Not applicable*, *Unknown*, *Asked but unknown*, *Temporarily unavailable*, *Not asked*, *Not available*, *Sufficient quantity*, and *Trace*. Null flavors are defined in the root level of the schema as ‘global lookup options’ and are automatically reused throughout the schema whenever the LookupOne or LookupMany value types are used. The value types LookupOne_NoGlobals and LookupMany_NoGlobals can be used if the null flavors should not be included in a particular lookup list.

Because concept definitions and classification schemas are subject to change over time due to progressive insight, FAIR Genomes users can choose to use lookup lists such as HPO terms in a static or in a dynamic way. A static solution refers to using an exact copy of a list, whereas a dynamic solution points to a source and keeps itself up-to-date at runtime. Both of these approaches have pros and cons. Static is easier to manage but may not be up-to-date, whereas dynamic is more difficult to manage but always up-to-date. Neither will affect interoperability greatly, but the choice of static versus dynamic lists should be made based on user requirements.

The FAIR Genomes schema contains a few circular dependencies. For instance, the *Material used in diagnosis* element in the *Clinical* module refers to a *Material*, indicating that this diagnosis or clinical examination is based on one or more sampled materials. Conversely, the *Belongs to diagnosis* element in *Material* refers to *Clinical*, indicating that one or multiple diagnoses were established based on reusing the same non-tumor material as a reference. A more straightforward example is a *Study* containing multiple people (i.e. instances of *Personal*), while people can also be part of multiple studies. However, circular dependencies cannot always be imported directly into database systems because, no matter the import order, there will be a reference to a table that does not exist yet. Until solutions are available (i.e. separating out all references and adding them at the end), we suggest altering relational database structures after importing the FAIR Genomes to complete the full schema.

### Developing interoperable solutions and resources

#### Automated schema to practice

We developed software to parse the schema and generate outputs including ‘blueprints’ that can be used to prepare EDCs for data entry, semantic resources, including an application ontology and various documentation formats. All of these outputs can be used in any desired combination to support a diverse IT landscape. Updating the outputs can be done at the push of a button instead of requiring development time every time the schema is updated. The time saved by maintaining these outputs can instead be used to increase the quality and completeness of the underlying schema. The advantages of using a generator design pattern are that it: (i) immediately builds and updates all outputs, (ii) ensures that all outputs remain synchronized and therefore interoperable (a critical aspect of FAIRification) and (iii) allows creation of additional generators when new types of outputs are required. The outputs that are available in release v1.1 are described in the next sections. An overview of how these outputs are created from the schema can be seen in Fig. 2.

Software was developed in Java to parse the FAIR Genomes YAML schema and provide this information to a range of output generators. Each output type (e.g. Markdown) has its own generator of which the class (e.g. ‘ToMarkdown.java’) is based on an abstract class called AbstractGenerator. This abstract class defines properties and behavior that are shared by all generators, such as line endings and checking the output folder. The software was built by Java SDK 11.0.8 and uses the following dependencies, managed by a Maven POM file: Apache POI 3.17, Apache POI OOXML 3.17, FasterXML Jackson 2.12.0, Eclipse RDF4J 3.5.1 and SnakeYAML 1.27.

#### Blueprints for data-capture systems

The blueprint for the MOLGENIS EDC is generated in MOLGENIS-EMX format that can be imported manually or automatically via an installation script. The script consists of MOLGENIS Commander (https://pypi.org/project/molgenis-commander) statements and imports all necessary tables, lookups and settings to a running MOLGENIS server as MOLGENIS-EMX files. A file named ‘sys_md_Package’ defines the the ‘fair-genomes’ database namespace. First, all attribute definitions for lookup tables are uploaded into ‘fair-genomes’. Second, all lookup data are imported into the database. Third, the actual table definitions based on the modules are added (i.e. *Personal*, *Clinical*, etc.). To finalize the full application, a custom home page is added with visual hyperlinks to each module and anonymous users are granted the rights to view and edit data. Supplementary Data S3 provides a detailed manual on how to install FAIR Genomes on a MOLGENIS Docker image, which includes running this import script. For MOLGENIS-EMX2 format, molgenis.csv is the file that defines the module and lookup table structures and attributes. The other CSV files in the generated molgenis-emx2 folder contain the lookup values. MOLGENIS-EMX2 files can be imported together as a ZIP file into a MOLGENIS-EMX2 database.

The ART-DECOR XML is created by an intermediate step. First, a codebook is created that consists of INFO.tsv, CODEBOOK.tsv and all lookup lists in TSV format. The INFO file contains the version, date, name and description of the schema. The CODEBOOK file contains the schema structure including modules, elements, data types and ontology references. The lookup lists are similar to the inputs, except for restructured headers. These files have language metadata and contain English values (value_en) and descriptions (description_en) that could be expanded to include other languages as well. This codebook is then translated into ART-DECOR® XML by ‘Excel codebook To ART-DECOR XML’ (https://github.com/aderidder/PALGACodebookToXML) by Sander de Ridder under GNU General Public License v3.0. The source code of this tool was incorporated here with an added TSV extension for the ‘Codebook’ class that allows this tool to use TSV files created earlier, in addition to supporting Excel-based codebooks. Other source code adaptations include the removal of GUI components. In future releases, we plan to refactor the ‘Codebook To ART-DECOR’ software into a Java library so that a common core can be reused and updated for both projects and possibly benefit other projects that have a need to automate ART-DECOR® XML creation.

iCRF Generator retrieves ART-DECOR projects hosted by Nictiz and provides a wizard to build EDC forms. Future updates of iCRF Generator will address potential import failures with the forms generated for Castor caused by long description fields. In addition, iCRF will be updated in the future to support LibreClinica (https://www.libreclinica.org), the community edition of OpenClinica.

A number of EDCs have added support for FAIR Data Point^65^ (FDP). In MOLGENIS-EMX, an FDP endpoint can be created by following the guide at https://molgenis.gitbooks.io/molgenis/content/guide-fair.html. For MOLGENIS-EMX2, FDP is also supported by importing or creating the proper definitions for Repository, Catalog, Dataset and Distribution from an Excel template (available at https://molgenis.github.io/molgenis-emx2, section *FAIR Data Point*). The FDP of Castor EDC includes catalogues, datasets, and distributions of Studies and can be browsed at https://fdp.castoredc.com/fdp.

#### Application ontology

The application ontology is available in the Turtle (TTL) format. The core schema file (fair-genomes.ttl) represents the definition of all the modules and elements in the FAIR Genomes schema. In addition, there is one TTL file for each unique lookup list, resulting in 29 TTL files in total for release v1.1. We successfully tested the conversion of TTL to ten other RDF formats including OWL using OntologyConverter v1.0 (https://github.com/sszuev/ont-converter).

Modules were defined as owl:Class and elements as owl:DatatypeProperty. Elements have values for rdfs:label, rdfs:domain, rdfs:isDefinedBy, dc:description and rdfs:range. Lookup options have values for rdfs:label, dc:description and rdfs:isDefinedBy, and are typed by the ‘a’ predicate.

We defined new terms as RDF-XML fragments that are individually hosted and accessible via the FAIR Genomes GitHub repository. These fragments contain semantic definitions for specific terms. New elements terms have values for rdf:Description, rdf:type, rdfs:label, rdfs:isDefinedBy, rdfs:domain, dc:description and dc:identifier. New lookup terms have values for rdf:Description, rdf:type, rdfs:label, rdfs:isDefinedBy, dc:description and dc:identifier. Via an XSLT stylesheet transformation, these fragments are rendered into a human-readable summary.

#### Documentation

A full overview of the FAIR Genomes schema has been created in the Markdown (.md) format. This format is typically rendered as human-readable HTML pages in the browser by platforms such as GitHub. As an HTML document, it supports hyperlinks for easy navigation within the document as well as directly linking to the ontologies and lookup lists used.

The LODE^60^ page was created using the online LODE web tool at https://essepuntato.it/lode/. The stand-alone local installation of LODE (available at https://github.com/essepuntato/LODE) has also been tested to ensure that it correctly generates the desired documentation output, in case the web tool would be unavailable. The generated core schema application ontology generated (fair-genomes.ttl) was used as input without any modification. The option ‘OWLAPI’ must be ticked both for online and local running of LODE by adding *owlapi = true* to the request URL. The lookup list TTL files were simply too big to be integrated into the LODE documentation directly. To include them in this documentation, hyperlinks to the lookup lists are listed under ‘Contributors’, which we acknowledge is semantically wrong and should be addressed in future releases. The LODE page output is saved and hosted on GitHub to prevent unnecessary burden on the LODE web service.

The LaTeX document output is created as a default documentclass ‘article’ with UTF-8 character set and no further package dependencies. The modules and elements are produced in a basic ‘table’ and ‘tabular’ structure. Conversion from LaTeX to PDF is performed by a Shell script using the commands ‘latex’, ‘dvips’ and ‘ps2pdf’ which must be installed for the script to function.

#### Published resources

The ART-DECOR® XML was imported as a project into the Nictiz hosting platform version 2.0, ART v2.0.18, DECOR core v2.0.17. It can be accessed by iCRF Generator^58^ to create case report forms for several major EDCs. It is available under project ID/OID 2.16.840.1.113883.2.4.3.11.60.120. In future updates, we plan to reuse lookups that are already present in the ART-DECOR® platform such as HPO, instead of including these ontologies within FAIR Genomes in order to reduce size and increase harmonization with other ART-DECOR® projects.

The BioPortal submission was prepared by converting the core FAIR Genomes application ontology from TTL to an OWL file using OntologyConverter v1.0. This OWL file was uploaded to https://bioportal.bioontology.org/ontologies/FG. The W3ID redirect was setup by forking https://github.com/perma-id/w3id.org, adding a *fair-genomes* folder containing a*.htaccess* file with redirect rules for Apache Web Server software. These additions were accepted as a pull request into the main w3id.org repository merged on April 12, 2021.

### Prototype systems

The FAIR Genomes MOLGENIS public demonstrator and production instance are created for MOLGENIS 8.6.2 and hosted on virtual machines with the following specifications: 2 CPU cores, 4GB RAM, 25GB disk, running CentOS 8, Apache Tomcat 9.0.39, Elasticsearch 5.6.16, PostgreSQL 11.9, nginx 1.14.1 and openjdk 11.0.9.1 2020-11-04 LTS.

The RD3 database for SolveRD runs on MOLGENIS 8.6.3 built on 2021-02-19 15:25 UTC. The content of this database is sensitive and currently only accessible to authorized users. Access may be requested via the appropriate Data Access Committee. However, public query options on aggregate level are available via Discovery Nexus, which is part of Cafe Variome^75^. Discovery Nexus allows users to retrieve (i) the number of samples matching specific criteria and (ii) sample identifiers from these search results. These identifiers may then be used to retrieve sample metadata from RD3 if the user is authorized.

The University Medical Center Groningen (UMCG) COSAS database will be the FAIR Genomes-compliant sample database at the UMCG Department of Genetics. Development is open source at https://github.com/molgenis/molgenis-cosas. Currently, prototypes are being developed based on MOLGENIS 8.6.3 using synthetic data. The data model is defined using YAML files, which may be converted to EMX using R scripts.

## Supplementary information

Supplementary Data S1

Supplementary Data S2

Supplementary Data S3

Supplementary Data S4

Supplementary Data S5

Supplementary Data S6

Supplementary Data S7

## Data Availability

The datasets generated and/or analyzed during the current study are available in the ‘FAIR Genomes’ repository, https://github.com/fairgenomes and at Zenodo^76^.

## Code availability

The FAIR Genomes code is available on GitHub at https://github.com/fairgenomes as Free and Open Source Software. The FAIR Genomes semantic metadata schema release v1.1 as described in this manuscript is available at https://github.com/fairgenomes/fairgenomes-semantic-model/tree/v1.1. The codebase is also available as an archive at Zenodo^76^. Both code and schema are released under a CC BY 4.0 license.
