# Supplementary material for: FAIR Genomes metadata schema promoting Next Generation Sequencing data reuse in Dutch healthcare and research
Source: Sci Data. 2022 Apr 13;9:169. doi: 10.1038/s41597-022-01265-x (PMC9008059; doi:10.1038/s41597-022-01265-x)
Supplement: Supplementary file 1 — Supplementary Data S1 [file 41597_2022_1265_MOESM1_ESM.pdf]

Supplementary Data S1:
FAIR Genomes Consortium members

Gurnoor Singh[1], XiaoFeng Liao[1], Rajaram Kaliyaperumal[6], Lisenka Vissers[1], Jeroen van Reeuwijk[1], Saskia Hiltemann[7], Jasmin Böhmer[3], Marissa de Valck[2,3], Lennart F. Johansson[2], Nienke van der Stoep[6], Daoud Sie[4], Marjan Weiss[1], Geert Frederix[3], Marco Roos[6], Erik van Iperen[8], Florien Gorter[1], Terry Vrijenhoek[3], Folkert W. Asselbergs[3], Sander van den Hoek[2], Joris van Montfrans[3], Rolf Sijmons[2], Hanneke W.M. van Deutekom[3], Pieter Neerincx[2], Fernanda de Andrade[2], Anna Niehues[1], Hindrik H.D. Kerstens[10], Annika Jacobsen[6], Katy Wolstencroft[6,14], Ies Nijman[3], Marcel Nelen[1], Ariaan Siezen[1], Koen ten Hove[1], Nine Knoers[2], Christian Gilissen[1], Sander de Ridder[4], Hans Scheffer[1], Stefan Willems[2], Wendy van Zelst-Stams[1], Helger Ijntema[1], Kim Elsink[3], Bart de Koning[9], Bauke Ylstra[4], Erik Sistermans[4], Patrick Kemmeren[10], Ina Geurts-Giele[7], Henne Holstege[4], Christine Staiger[11], Bastiaan Tops[10], Susanne Rebers[12], David van Zessen[7], Valesca Retèl[12], Edwin Cuppen[13], Peter van Tintelen[3], Esther van Enckevort[2], Lieneke Steeghs[1], Salome Scholtens[2], Leon Mei[6], Cor Oosterwijk[5], Andrew Stubbs[7], Jeroen Laros[6], Jeroen A.M. Beliën[4], Daphne Stemkens[5], K. Joeri van der Velde[2], Peter A.C. ‘t Hoen[1], Mariëlle E. van Gijn[2] and Morris A. Swertz[2].

[1] Radboud University Medical Center, Nijmegen, The Netherlands
[2] University Medical Center Groningen, The Netherlands
[3] University Medical Center Utrecht, The Netherlands
[4] Amsterdam UMC, Vrije Universiteit Amsterdam, Amsterdam, The Netherlands
[5] VSOP - Dutch Patient Alliance for Rare and Genetic Diseases
[6] Leiden University Medical Center, The Netherlands
[7] Erasmus Medical Center, Rotterdam, The Netherlands
[8] Durrer Center for Cardiovascular Research, Utrecht, The Netherlands
[9] Maastricht University Medical Center, The Netherlands
[10] Princess Máxima Center for Pediatric Oncology, Utrecht, The Netherlands
[11] Dutch Techcentre for Life Sciences, Utrecht, The Netherlands
[12] Netherlands Cancer Institute, Amsterdam, The Netherlands
[13] Hartwig Medical Foundation, Amsterdam, The Netherlands
[14] Leiden Institute for Advanced Computer Science, Leiden University, Leiden, The Netherlands
