# Supplementary material for: FAIR Genomes metadata schema promoting Next Generation Sequencing data reuse in Dutch healthcare and research
Source: Sci Data. 2022 Apr 13;9:169. doi: 10.1038/s41597-022-01265-x (PMC9008059; doi:10.1038/s41597-022-01265-x)
Supplement: Supplementary file 2 — Supplementary Data S2 [file 41597_2022_1265_MOESM2_ESM.pdf]

Supplementary Data S2:
FAIR Genomes semantic metadata schema

The FAIR Genomes semantic metadata schema to power reuse of NGS data in research and healthcare. Version 1.1-Minor, 2021-07-20. This model consists of **9 modules** that contain **110 metadata elements** and **85307 lookups** in total (excluding null flavors).

Module overview

Name	Description	Ontology	Nr. of elements
Study	A detailed examination, analysis, or critical inspection of one or multiple subjects designed to discover facts.	NCIT:C63536	9
Personal	Data, facts or figures about an individual; the set of relevant items would depend on the use case.	NCIT:C90492	12
Leaflet and consent form	A document explaining all the relevant information to assist an individual in understanding the expectations and risks in making a decision about a procedure. This document is presented to and signed by the individual or guardian.	NCIT:C16468	9
Individual consent	Consent given by a patient to a surgical or medical procedure or participation in a study, examination or analysis after achieving an understanding of the relevant medical facts and the risks involved.	NCIT:C16735	12
Clinical	Findings and circumstances relating to the examination and treatment of a patient.	NCIT:C25398	20

Name	Description	Ontology	Nr. of elements
Material	A natural substance derived from living organisms such as cells, tissues, proteins, and DNA.	NCIT:C43376	16
Sample preparation	A sample preparation for a nucleic acids sequencing assay.	OBI:0001902	9
Sequencing	The determination of complete (typically nucleotide) sequences, including those of genomes (full genome sequencing, de novo sequencing and resequencing), amplicons and transcriptomes.	EDAM:topic_3168	12
Analysis	An analysis applies analytical (often computational) methods to existing data of a specific type to produce some desired output.	EDAM:operation_2945	11

Module: Study

A detailed examination, analysis, or critical inspection of one or multiple subjects designed to discover facts. Ontology: NCIT:C63536.

Element	Description	Ontology	Values
Identifier	A unique proper name or character sequence that identifies this particular study.	OMIABIS:0000006	UniqueID
Name	A name that designates this study.	OMIABIS:0000037	String
Description	A statement or piece of writing that provides details on this study.	OMIABIS:0000036	Text

Element	Description	Ontology	Values
Inclusion criteria	The conditions which, if met, make an person eligible for participation in this study.	OBI:0500027	InclusionCriteria lookup (14 choices of type)
Principal investigator	The principle investigator or responsible person for this study.	OMIABIS:0000100	String
Contact information	An email address for the purpose of contacting the study contact person.	OMIABIS:0000035	String
Study design	A plan specification comprised of protocols (which may specify how and what kinds of data will be gathered) that are executed as part of this study.	OBI:0500000	Text
Start date	The date on which this study began.	NCIT:C69208	Date
Completion date	The date on which the concluding information for this study is completed. Usually, this is when the last subject has a final visit, or the main analysis has finished, or any other protocol-defined completion date.	NCIT:C142702	Date

Module: Personal

Data, facts or figures about an individual; the set of relevant items would depend on the use case. Ontology: NCIT:C90492.

Element	Description	Ontology	Values
Personal identifier	A unique proper name or character sequence that identifies this particular person.	NCIT:C164337	UniqueID
Phenotypic sex	An organismal quality inhering in a bearer by virtue of the bearer's physical expression of sexual characteristics.	PATO:0001894	PhenotypicSex lookup (4 choices of type)
Genotypic sex	A biological sex quality inhering in an individual based upon genotypic composition of sex chromosomes.	PATO:0020000	GenotypicSex lookup (12 choices of type)
Country of residence	Country of residence at enrollment.	NCIT:C171105	Countries lookup (249 choices of type)
Ancestry	Population category defined using ancestry informative markers (AIMs) based on genetic/genomic data.	NCIT:C176763	Ancestry lookup (305 choices of type)
Country of birth	The country that this person was born in.	GENEPIO:0001094	Countries lookup (249 choices of type)
Year of birth	The year in which this person was born.	NCIT:C83164	Integer
Inclusion status	An indicator that provides information on the current health status of this person.	NCIT:C166244	InclusionStatus lookup (4 choices of type)
Age at death	The age at which death occurred.	NCIT:C135383	Integer
Primary affiliated institute	The most significant institute for medical consultation and/or	NCIT:C25412	Institutes lookup (218 choices of type)

Element	Description	Ontology	Values
	study inclusion in context of the genetic disease of this person.		
Resources in other institutes	Material or data related to this person that is not captured by this system though known to be available in other institutes such as biobanks or hospitals.	NCIT:C19012	Institutes lookup (218 choices of type)
Participates in study	Reference to the study or studies in which this person participates.	RO:0000056	Reference to instances of Study

Module: Leaflet and consent form

A document explaining all the relevant information to assist an individual in understanding the expectations and risks in making a decision about a procedure. This document is presented to and signed by the individual or guardian.
Ontology: NCIT:C16468.

Element	Description	Ontology	Values
Leaflet title	A title or name given to the leaflet that belongs to this consent form.	DC:title	String
Leaflet date	A point or period of time associated with the publication of this leaflet that belongs to this consent form.	DC:date	Date
Leaflet version	The version, edition, or adaptation of this leaflet that belongs to this consent form.	DC:hasVersion	String
Consent form identifier	A unique proper name or character sequence that identifies this particular leaflet and consent form combination used in signing individual consent. Using a DOI would be optimal. Using any	DC:identifier	UniqueID

Element	Description	Ontology	Values
	resolvable URL is suboptimal but still preferable over using a plain text value.		
Consent form title	A title or name given to this consent form.	DC:title	String
Consent form accepted date	Date of acceptance of this consent form.	DC:dateAccepted	Date
Consent form valid until	End date of the validity of this consent form.	DC:valid	Date
Consent form creator	Indicates the authoritative body who brought this consent form into existence.	NCIT:C42628	Institutes lookup (218 choices of type)
Consent form version	The version, edition, or adaptation of this consent form.	DC:hasVersion	String

Module: Individual consent

Consent given by a patient to a surgical or medical procedure or participation in a study, examination or analysis after achieving an understanding of the relevant medical facts and the risks involved. Ontology: NCIT:C16735.

Element	Description	Ontology	Values
Individual consent identifier	A unique proper name or character sequence that identifies this particular signed individual consent.	ICO:0000044	UniqueID

Element	Description	Ontology	Values
Person consenting	Reference to the person (i.e. subject) to whom this individual consent applies.	IAO:0000136	Reference to instances of Personal
Consent form used	Reference to the informed consent form that was signed. Points to a particular instance of leaflet and consent form that usually exists as a record (i.e. a row) within the same database as this individual consent.	IAO:0000136	Reference to instances of Leaflet and consent form
Collected by	Indicates the institute who performed the collection act.	NCIT:C45262	Institutes lookup (218 choices of type)
Signing date	A date specification that designates when this individual consent form was signed.	ICO:0000036	Date
Valid from	Starting date of the validity of this individual consent.	DC:valid	Date
Valid until	End date of the validity of this individual consent.	DC:valid	Date
Represented by	An individual who is authorized under applicable State or local law to consent on behalf of a child or incapable person to general medical care including participation in clinical research.	NCIT:C142600	RepresentedBy lookup (3 choices of type)
Data use permissions	A data item that is used to indicate consent permissions for datasets and/or materials, and relates to the purposes	DUO:0000001	DataUsePermissions lookup (5 choices of type)

Element	Description	Ontology	Values
	for which datasets and/or material might be used.		
Data use modifiers	Data use modifiers indicate additional conditions for use. For instance, a dataset is restricted to investigations into specific diseases or performed at specific geographical locations.	DUO:0000017	DataUseModifiers lookup (23 choices of type)
Data use specification	Further specification of applied data use permissions and modifiers. For example, a list of countries in case of geographic restrictions or a list of diseases when restricted to disease-specific research.	SIO:000090	Text
Allow recontacting	The procedure of recontacting the patient for specified reasons. This means the patient agrees to be re-identifiable under those circumstances.	NCIT:C25737	Recontacting lookup (3 choices of type)

Module: Clinical

Findings and circumstances relating to the examination and treatment of a patient. Ontology: NCIT:C25398.

Element	Description	Ontology	Values
Clinical identifier	A unique proper name or character sequence that identifies this particular clinical examination.	NCIT:C87853	UniqueID

Element	Description	Ontology	Values
Belongs to person	Reference to the person whom this clinical information is about.	IAO:0000136	Reference to instances of Personal
Phenotype	The outward appearance of the individual. In medical context, these are often the symptoms caused by a disease.	NCIT:C16977	Phenotypes lookup (15802 choices of type)
Unobserved phenotype	Phenotypes or symptoms that were looked for but not observed, which may help in differential diagnosis or establish incomplete penetrance.	HL7:C0442737	Phenotypes lookup (15802 choices of type)
Phenotypic data available	Types of phenotypic data collected in a clinical setting that is potentially available upon request.	NCIT:C15783	DCMITypes lookup (6 choices of type)
Clinical diagnosis	A diagnosis made from a study of the signs and symptoms of a disease.	NCIT:C15607	Diseases lookup (9700 choices of type)
Molecular diagnosis gene	Gene affected by pathogenic variation that is causal for disease of the patient.	NCIT:C20826	Genes lookup (19202 choices of type)
Molecular diagnosis other	Causal variant in HGVS notation with optional classification or free text explaining any other molecular mechanisms involved.	NCIT:C20826	Text

Element	Description	Ontology	Values
Age at diagnosis	The age, measured from some defined time point (e.g. birth) at which a patient is diagnosed with a disease.	SNOMEDCT:423493009	Integer
Age at last screening	Age of the patient at the moment of the most recent screening.	NCIT:C81258	Integer
Medication	A drug product that contains one or more active and/or inactive ingredients used by the patient intended to treat, prevent or alleviate the symptoms of disease.	NCIT:C459	Drugs lookup (5632 choices of type)
Drug regimen	The specific way a therapeutic drug is to be taken, including formulation, route of administration, dose, dosing interval, and treatment duration.	NCIT:C142516	Text
Family members affected	Family members related by descent rather than by marriage or law who were diagnosed with the same condition as the individual who is the primary focus of investigation (i.e. the proband).	HP:0032320	FamilyMembers lookup (41 choices of type)
Family members sequenced	Family members related by descent rather than by marriage or law who were also tested by	NCIT:C79916	FamilyMembers lookup (41 choices of type)

Element	Description	Ontology	Values
	next-generation sequencing.		
Consanguinity	Information on whether the patient is a child from two family members who are second cousins or closer.	GSSO:007578	String
Medical history	A record of a person's background regarding health, occurrence of disease events and surgical procedures.	NCIT:C18772	MedicalHistory lookup (1154 choices of type)
Age of onset	Age of onset of clinical manifestations related to the disease of the patient.	Orphanet:C023	Integer
First contact	First contact of the patient with a specialised center in context of disease or study inclusion.	LOINC:MTHU048806	Date
Functioning	Patient's classification of functioning i.e. disability profile according to International Classification of Functioning and Disability (ICF).	NCIT:C21007	Text
Material used in diagnosis	This diagnosis c.q. clinical examination is based on one or more sampled materials.	SIO:000641	String

Module: Material

A natural substance derived from living organisms such as cells, tissues, proteins, and DNA. Ontology: NCIT:C43376.

Element	Description	Ontology	Values
Material identifier	A unique proper name or character sequence that identifies this particular material.	NCIT:C93400	UniqueID
Collected from person	Reference to the person from whom this material was collected.	SIO:000244	Reference to instances of Personal
Belongs to diagnosis	Reference to a diagnosis c.q. clinical examination of which this material may be a part of. There can be multiple diagnoses when a non-tumor material is reused as reference.	SIO:000068	Reference to instances of Clinical
Sampling timestamp	Date and time at which this material was collected.	EFO:0000689	DateTime
Registration timestamp	Date and time at which this material was listed or recorded officially, i.e. officially qualified or enrolled.	NCIT:C25646	DateTime
Sampling protocol	The procedure whereby this material was sampled for an analysis.	EFO:0005518	Text
Sampling protocol deviation	A variation from processes or procedures defined in the sampling protocol. Deviations usually do not preclude the overall evaluability of subject data for either efficacy or safety, and are often	NCIT:C50996	String

Element	Description	Ontology	Values
	acknowledged and accepted in advance by the sponsor.		
Reason for sampling protocol deviation	The rationale for why a deviation from the sampling protocol has occurred.	NCIT:C93529	String
Biospecimen type	The type of material taken from a biological entity for testing, diagnostic, propagation, treatment or research purposes.	NCIT:C70713	BiospecimenTypes lookup (403 choices of type)
Anatomical source	Biological entity that constitutes the structural organization of an individual member of a biological species from which this material was taken.	NCIT:C103264	AnatomicalSources lookup (13827 choices of type)
Pathological state	The pathological state of the tissue from which this material was derived.	GO:0001894	PathologicalState lookup (4 choices of type)
Storage conditions	The conditions under which this biological material was stored.	NCIT:C96145	StorageConditions lookup (26 choices of type)
Expiration date	The date beyond which this material is no longer regarded as fit for use.	NCIT:C164516	Date
Percentage tumor cells	The percentage of tumor cells compared to total cells present in this material.	NCIT:C127771	Decimal
Physical location	A place on the Earth where this material is located, by its name or by its geographical location.	GAZ:00000448	String

Element	Description	Ontology	Values
	This definition is intentionally vague to allow reuse locally (e.g. which freezer), for contacting (e.g. which institute), broadly for logistical or legal reasons (e.g. city, country or continent).		
Derived from	Indicate if this material was produced from or related to another.	NCIT:C28355	String

Module: Sample preparation

A sample preparation for a nucleic acids sequencing assay. Ontology: OBI:0001902.

Element	Description	Ontology	Values
Sampleprep identifier	A unique proper name or character sequence that identifies this particular sample preparation.	NCIT:C132299	UniqueID
Belongs to material	Reference to the source material from which this sample was prepared.	NCIT:C25683	Reference to instances of Material
Input amount	Amount of input material in nanogram (ng).	AFRL:0000010	Integer
Library preparation kit	Pre-filled, ready-to-use reagent cartridges intented to improve chemistry, cluster density and read length as well as improve quality (Q) scores for this sample. Reagent components are encoded to interact with the sequencing system to validate compatibility with user-defined applications.	GENEPIO:0000085	NGSKits lookup (616 choices of type)

Element	Description	Ontology	Values
PCR free	Indicates whether a polymerase chain reaction (PCR) was used to prepare this sample. PCR is a method for amplifying a DNA base sequence using multiple rounds of heat denaturation of the DNA and annealing of oligonucleotide primers complementary to flanking regions in the presence of a heat-stable polymerase.	NCIT:C17003	Boolean
Target enrichment kit	Indicates which target enrichment kit was used to prepare this sample. Target enrichment is a pre-sequencing DNA preparation step where DNA sequences are either directly amplified (amplicon or multiplex PCR-based) or captured (hybrid capture-based) in order to only focus on specific regions of a genome or DNA sample.	NCIT:C154307	NGSKits lookup (616 choices of type)
UMIs present	Indicates whether any unique molecular identifiers (UMIs) are present. An UMI barcode is a short nucleotide sequence that is used to identify reads originating from an individual mRNA molecule.	EFO:0010199	Boolean
Intended insert size	In paired-end sequencing, the DNA between the adapter sequences is the insert. The length of this sequence is known as the insert size, not to be confused with the inner distance between reads. So, fragment length equals read adapter length (2x) plus insert size, and insert size equals read lenght (2x) plus inner distance.	FG:0000001	Integer
Intended read length	The number of nucleotides intended to be ordered from each side of a nucleic acid fragment obtained after the completion of a sequencing process.	NCIT:C153362	Integer

Module: Sequencing

The determination of complete (typically nucleotide) sequences, including those of genomes (full genome sequencing, de novo sequencing and resequencing), amplicons and transcriptomes. Ontology: EDAM:topic_3168.

Element	Description	Ontology	Values
Sequencing identifier	A unique proper name or character sequence that identifies this particular nucleic acid sequencing assay.	NCIT:C171337	UniqueID
Belongs to sample	Reference to the prepared sample, i.e. the source that was sequenced.	NCIT:C25683	Reference to instances of Sample preparation
Sequencing date	Date on which this sequencing assay was performed.	GENEPIO:0000069	Date
Sequencing platform	The used sequencing platform (i.e. brand, name of a company that produces sequencer equipment).	GENEPIO:0000071	SequencingPlatform lookup (7 choices of type)
Sequencing instrument model	The used product name and model number of a manufacturer's genomic (dna) sequencer.	GENEPIO:0001921	SequencingInstrumentModels lookup (39 choices of type)
Sequencing method	Method used to determine the order	FIX:0000704	SequencingMethods lookup (35 choices of type)

Element	Description	Ontology	Values
	of bases in a nucleic acid sequence.		
Average read depth	The average number of times a particular locus (site, nucleotide, amplicon, region) was sequenced.	NCIT:C155320	Integer
Observed read length	The number of nucleotides successfully ordered from each side of a nucleic acid fragment obtained after the completion of a sequencing process.	NCIT:C153362	Integer
Observed insert size	In paired-end sequencing, the DNA between the adapter sequences is the insert. The length of this sequence is known as the insert size, not to be confused with the inner distance between reads. So, fragment length equals read adapter length (2x) plus insert size, and insert size equals read lenght (2x) plus inner distance.	FG:0000002	Integer
Percentage Q30	Percentage of reads with a Phred quality score over 30, which	GENEPIO:0000089	Decimal

Element	Description	Ontology	Values
	indicates less than a 1/1000 chance that the base was called incorrectly.		
Percentage TR20	Percentage of the target sequence on which 20 or more unique reads were successfully mapped.	FG:0000003	Decimal
Other quality metrics	Other NGS quality control metrics, including but not limited to (i) sequencer metrics such as yield, error rate, density (K/mm2), cluster PF (%) and phas/prephas (%), (ii) alignment metrics such as QM insert size, GC content, QM duplicated reads (%), QM error rate, uniformity/evenness of coverage and maternal cell contamination, and (iii) variant call metrics such as number of SNVs/CNVs/SVs called, number of missense/nonsense variants, common variants (%), unique variants (%), gender match and trio inheritance check.	EDAM:data_3914	Text

Module: Analysis

An analysis applies analytical (often computational) methods to existing data of a specific type to produce some desired output. Ontology: EDAM:operation_2945.

Element	Description	Ontology	Values
Analysis identifier	A unique proper name or character sequence that identifies this particular analysis.	AFR:0001979	UniqueID
Belongs to sequencing	Reference to the sequencing that was performed, i.e. the source on which this analysis was based.	NCIT:C25683	Reference to instances of Sequencing
Physical data location	A place on the Earth where the data is located, by its name or by its geographical location. This definition is intentionally vague to allow reuse locally (e.g. which computer), for contacting (e.g. which institute), broadly for logistical or legal reasons (e.g. city, country or continent).	GAZ:00000448	String
Abstract data location	The file location of the data, or a copy of the data, on an electronically accessible device for preservation (either in plain-text or encrypted format).	NCIT:C142494	String
Data formats stored	Which data file formats (i.e. defined ways or layouts of representing and structuring data in a computer file, blob, string, message, or elsewhere) are	NCIT:C142494	DataFormats lookup (582 choices of type)

Element	Description	Ontology	Values
	stored and potentially available.		
Algorithms used	Any used problem-solving procedures implemented in software to be executed by a computer.	NCIT:C16275	Text
Reference genome used	The specific build of the human genome used as reference for sequence alignment and variant calling.	EDAM:data_2340	GenomeAccessions lookup (29 choices of type)
Bioinformatic protocol used	A human-readable collection of information about about how a scientific experiment or analysis was carried out that results in a specific set of data or results used for further analysis or to test a specific hypothesis.	EDAM:data_2531	Text
Bioinformatic protocol deviation	A variation from processes or procedures defined in the bioinformatic protocol. Deviations usually do not preclude the overall evaluability of subject data for either efficacy or safety, and are often acknowledged and accepted in advance by the sponsor.	NCIT:C50996	String
Reason for bioinformatic protocol deviation	The rationale for why a deviation from the bioinformatic protocol has occurred.	NCIT:C93529	String

Element	Description	Ontology	Values
WGS guideline followed	Any followed systematic statement of policy rules or principles. Guidelines may be developed by government agencies at any level, institutions, professional societies, governing boards, or by convening expert panels.	NCIT:C17564	String

Null flavors

Each lookup in FAIR Genomes is supplemented with so-called 'null flavors' from HL7. These can be used to indicate precisely why a particular value could not be entered into the system, providing substantially more insight than simply leaving a field empty.

Value	Description	Ontology
NoInformation	The value is exceptional (missing, omitted, incomplete, improper). No information as to the reason for being an exceptional value is provided. This is the most general exceptional value. It is also the default exceptional value.	HL7:NI
Invalid	The value as represented in the instance is not a member of the set of permitted data values in the constrained value domain of a variable.	HL7:INV
Derived	An actual value may exist, but it must be derived from the provided information (usually an EXPR generic data type extension will be used to convey the derivation expression .	HL7:DER
Other	The actual value is not a member of the set of permitted data values in the constrained value domain of a variable.The actual value is not a member of the set of permitted data values in the constrained value domain of a variable. (e.g., concept not provided by required code system).	HL7:OTH
Negative infinity	Negative infinity of numbers.	HL7:NINF
Positive infinity	Positive infinity of numbers.	HL7:PINF
Un-encoded	The actual value has not yet been encoded within the approved value domain.	HL7:UNC

Value	Description	Ontology
Masked	There is information on this item available but it has not been provided by the sender due to security, privacy or other reasons. There may be an alternate mechanism for gaining access to this information.	HL7:MSK
Not applicable	Known to have no proper value (e.g., last menstrual period for a male).	HL7:NA
Unknown	A proper value is applicable, but not known.	HL7:UNK
Asked but unknown	Information was sought but not found (e.g., patient was asked but didn't know)	HL7:ASKU
Temporarily unavailable	Information is not available at this time but it is expected that it will be available later.	HL7:NAV
Not asked	This information has not been sought. (e.g., patient was not asked)	HL7:NASK
Not available	Information is not available at this time (with no expectation regarding whether it will or will not be available in the future).	HL7:NAVU
Sufficient quantity	The specific quantity is not known, but is known to be non-zero and is not specified because it makes up the bulk of the material. e.g. 'Add 10mg of ingredient X, 50mg of ingredient Y, and sufficient quantity of water to 100mL.' The null flavor would be used to express the quantity of water.	HL7:QS
Trace	The content is greater than zero, but too small to be quantified.	HL7:TRC
