# Supplementary material for: FAIR Genomes metadata schema promoting Next Generation Sequencing data reuse in Dutch healthcare and research
Source: Sci Data. 2022 Apr 13;9:169. doi: 10.1038/s41597-022-01265-x (PMC9008059; doi:10.1038/s41597-022-01265-x)
Supplement: Supplementary file 3 — Supplementary Data S3 [file 41597_2022_1265_MOESM3_ESM.pdf]

Supplementary Data S3:

FAIR Genomes MOLGENIS Docker

Command-line tool prerequisites

- Git client (see: https://git-scm.com/downloads)
- Docker client (see: https://docs.docker.com/get-docker)

Test if everything is installed correctly

`git --version` should return something like `git version 2.8.1`
`docker --version` should return something like `Docker version 20.10.5`

Clone MOLGENIS Docker and FAIR Genomes

```
git clone https://github.com/fairgenomes/fairgenomes-semantic-model.git
```

Bring MOLGENIS Docker online and load the fair genomes data

- Start MOLGENIS services in docker

```
cd fairgenomes-semantic-model/misc/molgenis/docker
docker-compose up -d
```

- Many files will be imported, to check the logs type:

```
docker-compose logs --follow commander
```

This process should look like

```
✓ Importing sys_md_Package.tsv
✓ Importing study_inclusioncriteria_attributes.tsv
✓ Importing personal_phenotypicsex_attributes.tsv
⠕ Importing personal_genotypicsex_attributes.tsv
```

All done!

- Start using FAIR Genomes by going to http://localhost/
- Anonymous users (i.e. not logged in) should already have edit/view rights
- To do and see absolutely everything, log in as `admin`, pw `admin`

Troubleshooting

To recreate everything, run `nuke.sh`

Alternative: How to manually install FAIR Genomes on a MOLGENIS Docker image

Command-line tool prerequisites

- Git client (see: https://git-scm.com/downloads)
- Docker client (see: https://docs.docker.com/get-docker)
- Python3 (see: https://www.python.org/downloads)
- pip3 (use e.g. `sudo apt-get -y install python3-pip`)
- MOLGENIS Commander (use e.g. `pip3 install molgenis-commander`)

Test if everything is installed correctly

`git --version` should return something like `git version 2.8.1`
`docker --version` should return something like `Docker version 20.10.5`
`python3 --version` should return something like `Python 3.7.2`
`pip3 --version` should return something like `pip 19.3.1`
`mcmd --version` should return something like `MOLGENIS Commander 1.10.3`

Clone MOLGENIS Docker and FAIR Genomes

```
git clone https://github.com/molgenis/docker.git
git clone https://github.com/fairgenomes/fairgenomes-semantic-model.git
```

Bring MOLGENIS Docker online

- Start your Docker client
- Then compose the MOLGENIS Docker image

```
cd docker/molgenis/8.7
docker-compose up
```

Connect MOLGENIS Commander to MOLGENIS Docker

- Either follow step-by-step setup instructions by running `mcmd`, or
- Edit `~/.mcmd/mcmd.yaml` file to include

```
  selected: http://localhost/
  auth:
  - url: http://localhost/
    username: admin
    password: admin
```

- If all is well, ping the server using `mcmd ping` which should return something like

```
Host:  http://localhost/
Status:  Online
Version:  8.7.2
User:  admin
```

Setup FAIR Genomes in the MOLGENIS Docker

- Go back to the directory where `git clone` was executed
- Step into the FAIR Genomes molgenis-emx folder and run the setup script

```
cd fairgenomes-semantic-model/generated/molgenis-emx/
sh setup.sh
```

- Many files will be imported. This process should look like

```
✔ Importing sys_md_Package.tsv
✔ Importing study_inclusioncriteria_attributes.tsv
✔ Importing personal_phenotypicsex_attributes.tsv
⠍ Importing personal_genotypicsex_attributes.tsv
```

All done!

- Start using FAIR Genomes by going to http://localhost/
- Anonymous users (i.e. not logged in) should already have edit/view rights
- To do and see absolutely everything, log in as `admin`, pw `admin`

Troubleshooting

- If you want to override existing imports, open the `~/.mcmd/mcmd.yaml` file and change `import_action: add` into `import_action: add_update_existing`
