# Supplementary material for: FAIR Genomes metadata schema promoting Next Generation Sequencing data reuse in Dutch healthcare and research
Source: Sci Data. 2022 Apr 13;9:169. doi: 10.1038/s41597-022-01265-x (PMC9008059; doi:10.1038/s41597-022-01265-x)

Supplementary Data S4:
FAIR Genomes MOLGENIS walkthrough

The MOLGENIS FAIR Genomes app can be used by visiting the FAIR Genomes public demo, by running the Docker install, or by performing a regular server install. See: https://molgenis.org and http://fairgenomes.org.

After launch, you are taken to the homepage. Start by clicking on a module such as Sequencing.

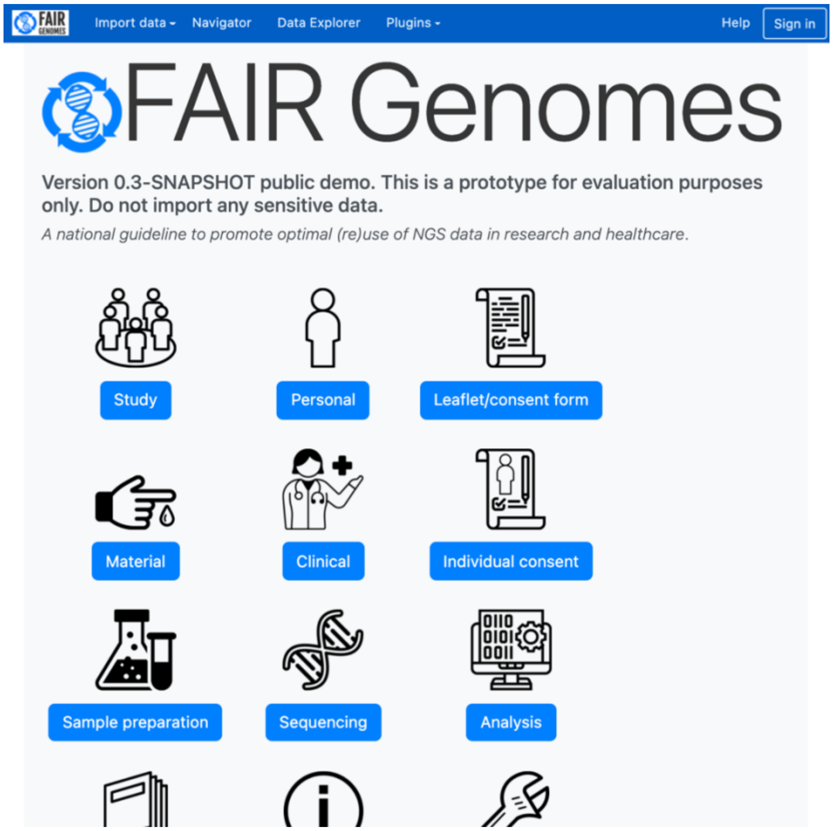

The Sequencing table is now shown in the Data Explorer. Here, you can select which columns you want to see, and apply filters to reduce the number of rows. Click the green [+] to create a new row.

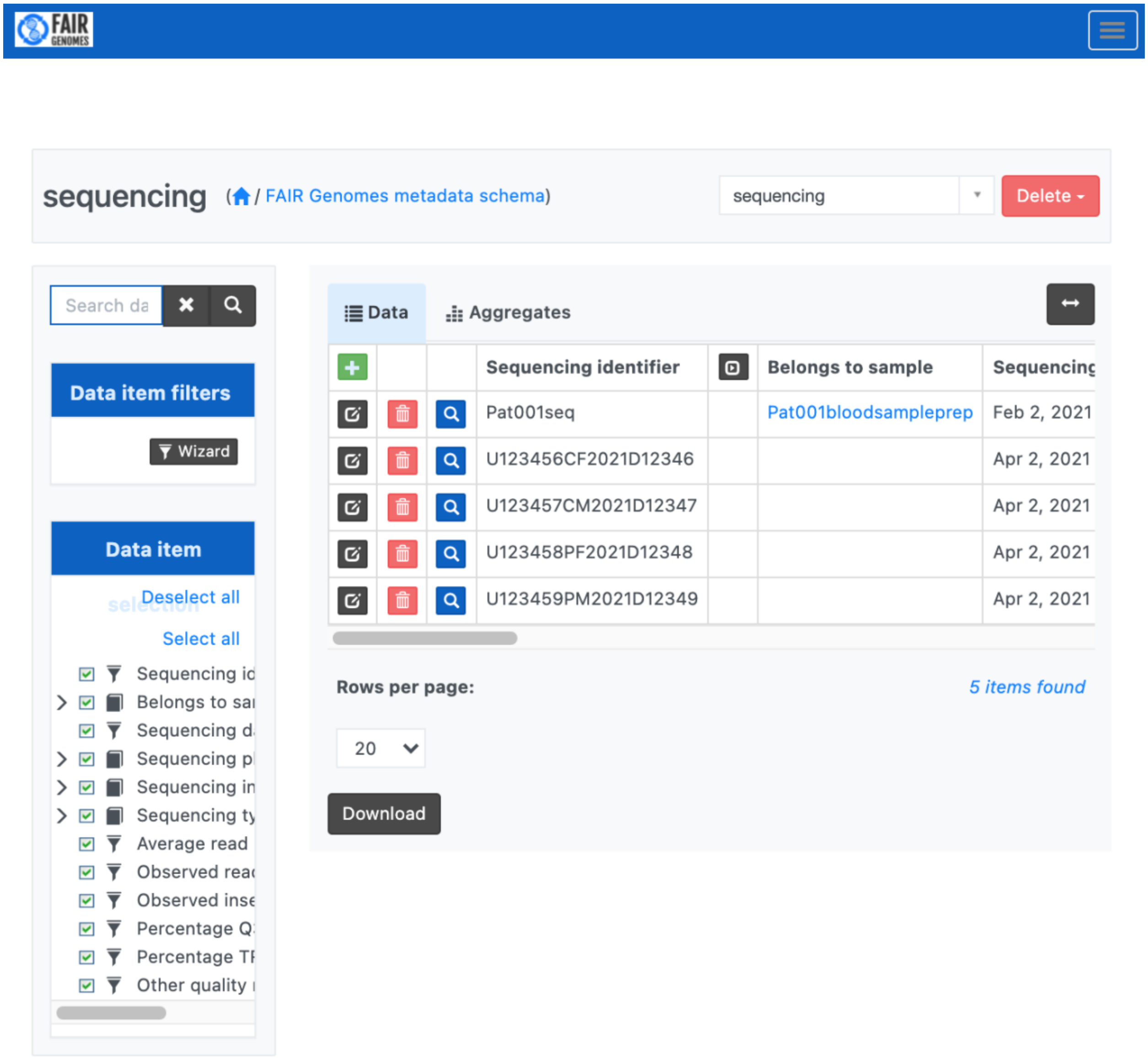

A form view is shown where values for a new row can be entered. Click Save when you are done.

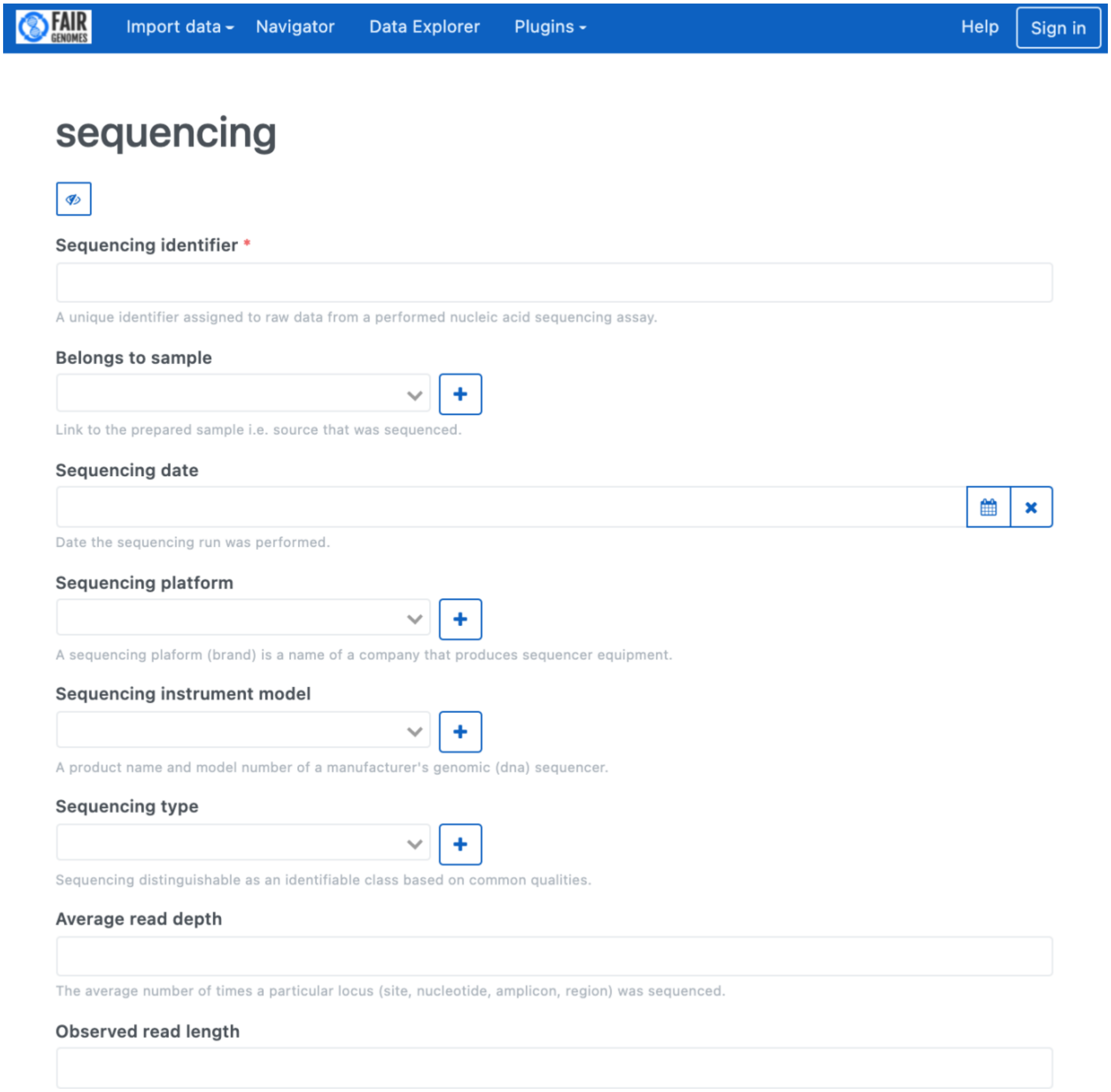

After saving, you are returned to the Data Explorer. The various modules in FAIR Genomes are linked to each other. You can click on hyperlinks to follow references in the database. For example, Sequencing is performed on a Sample. The details for the linked Sample can be viewed.

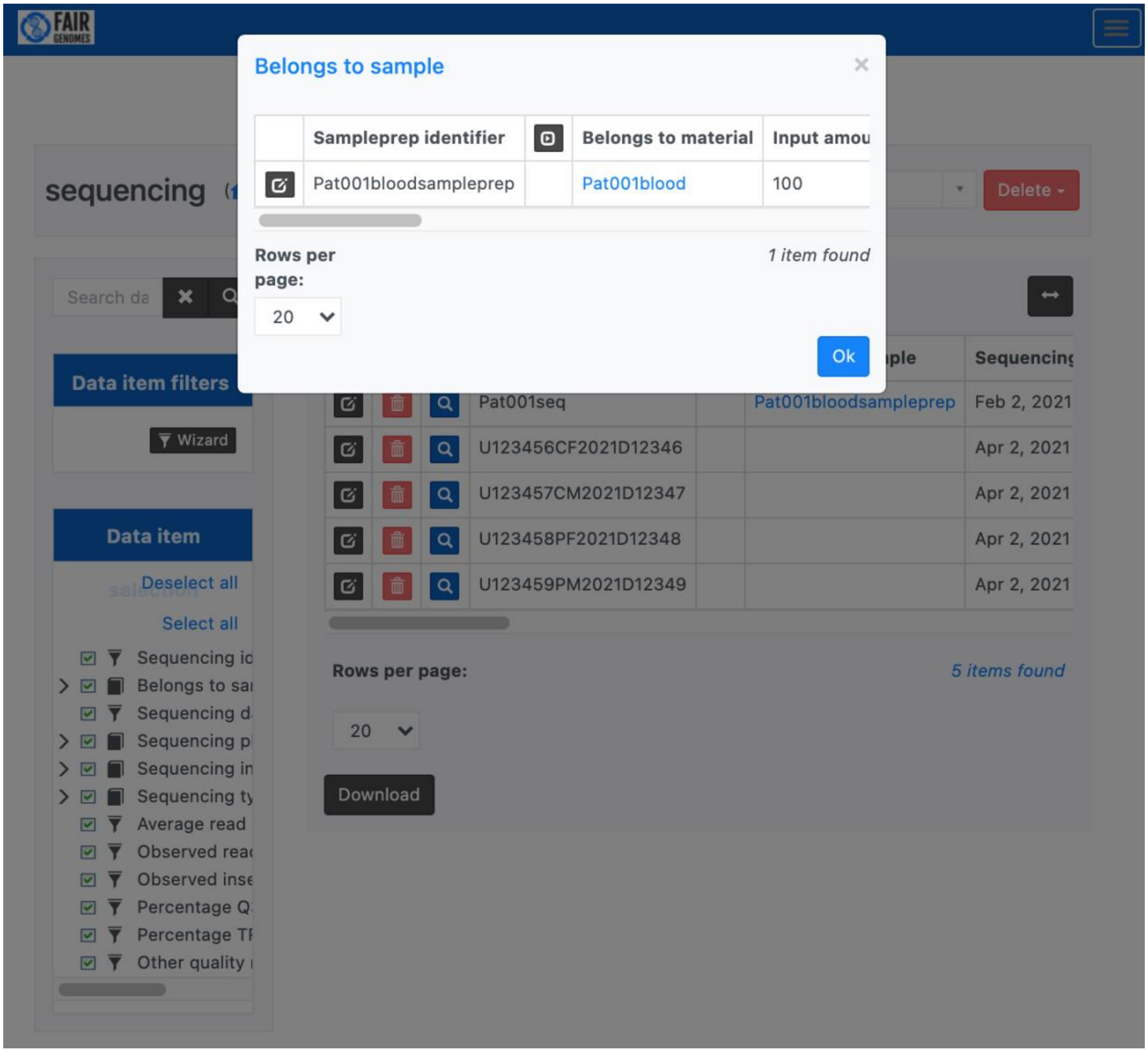

You can find all of the FAIR Genomes lookups, i.e. underlying ontological code systems, by clicking Lookups on homepage. This will take you to the Navigator view with all database tables. These underlying data can be modified if necessary.

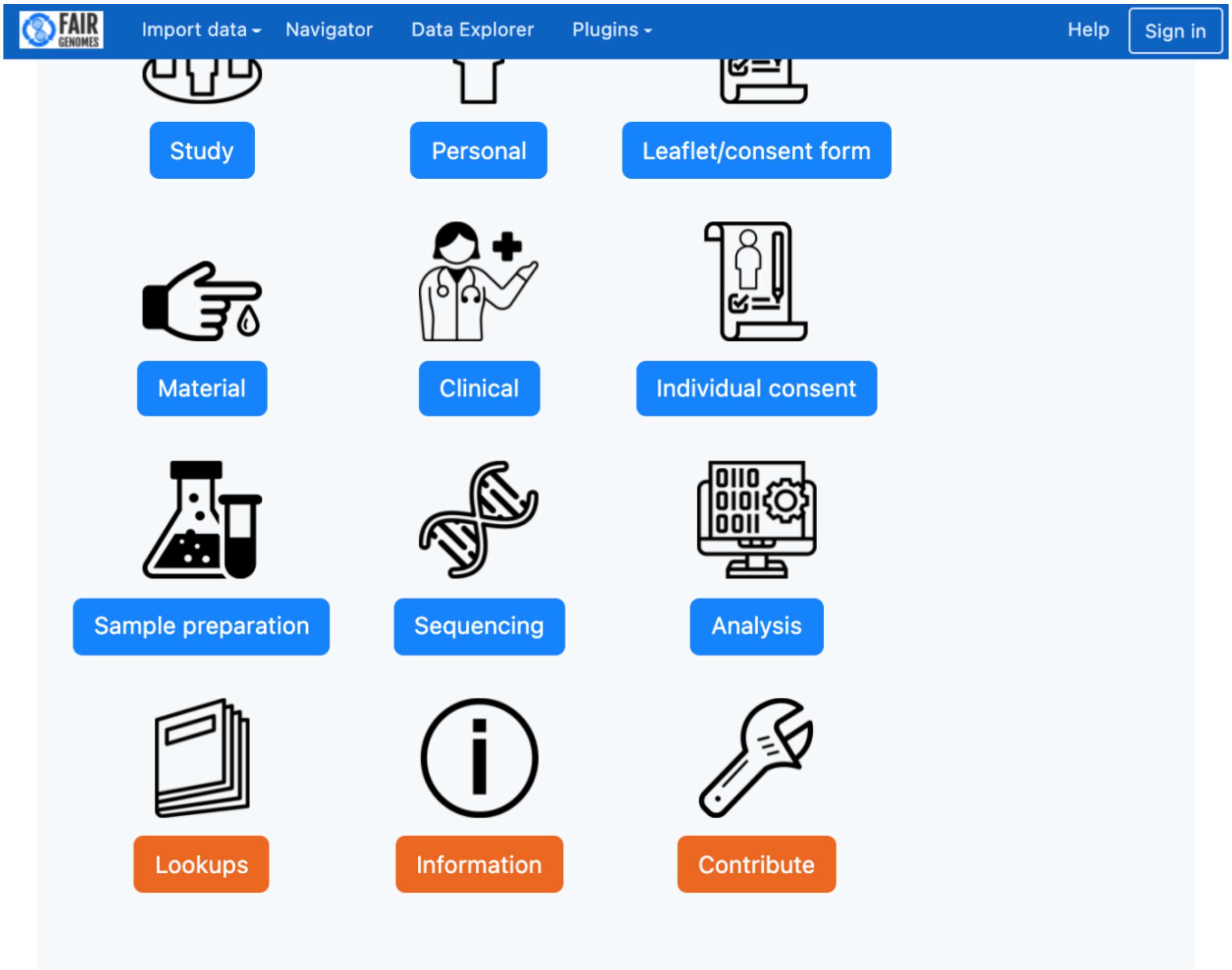

If you would like to perform a ‘Google search’, you can use the Search All function available via the Plugins menu.

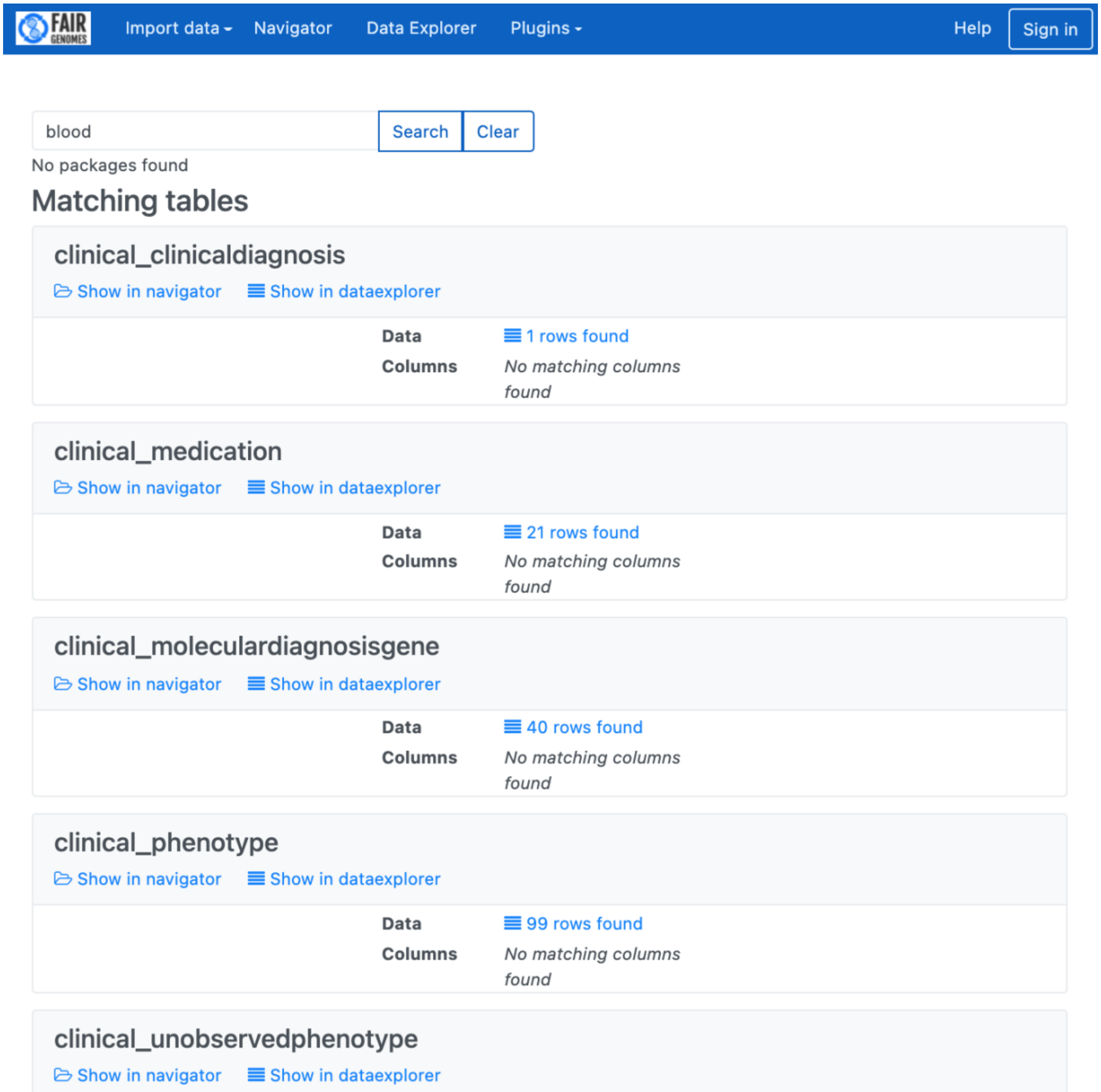

MOLGENIS offers powerful features such as altering the underlying data structure of the database itself. This can be done in the MetaData manager.

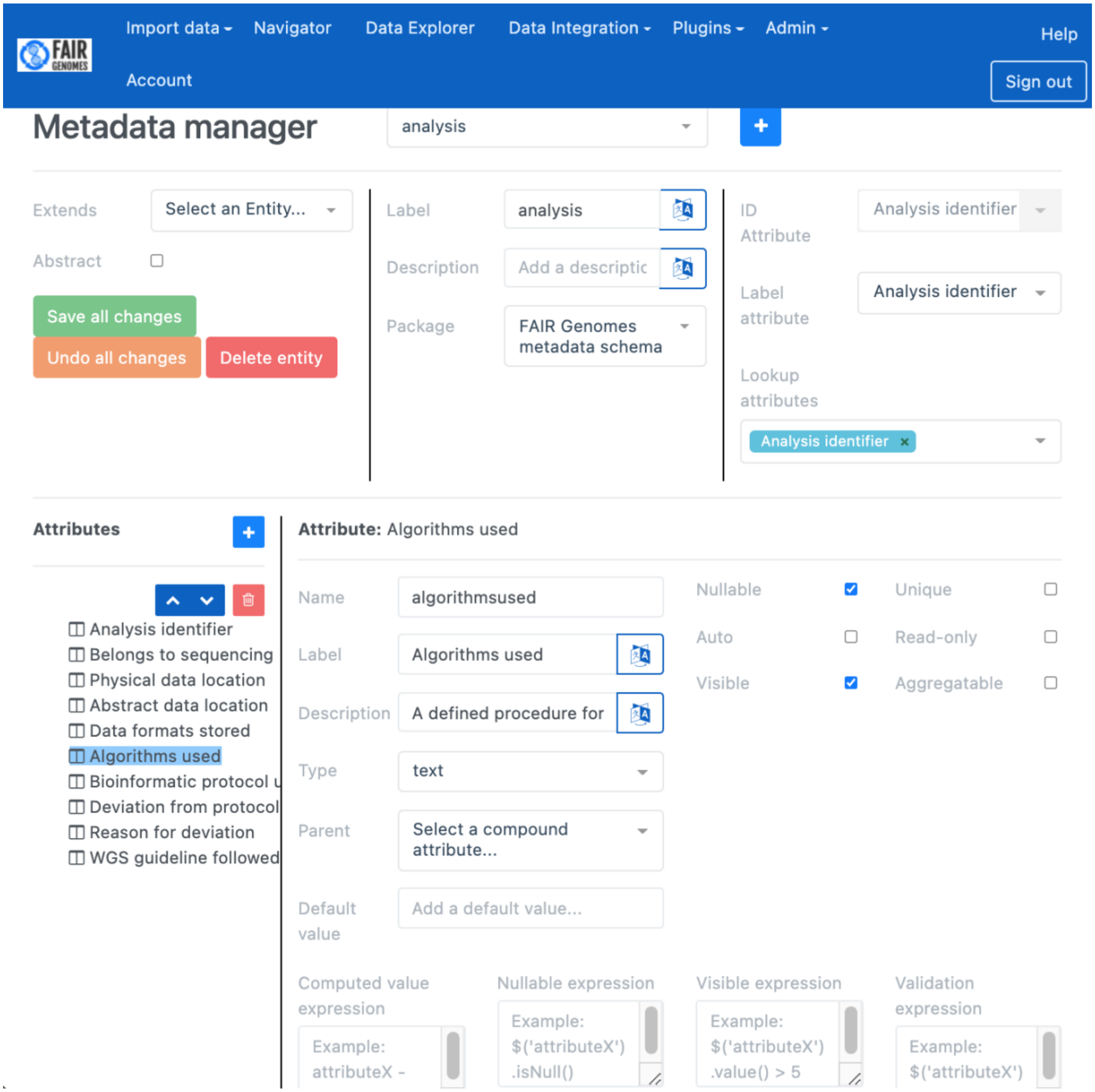

The menu structure of the user interface can also be adjusted to any needs.

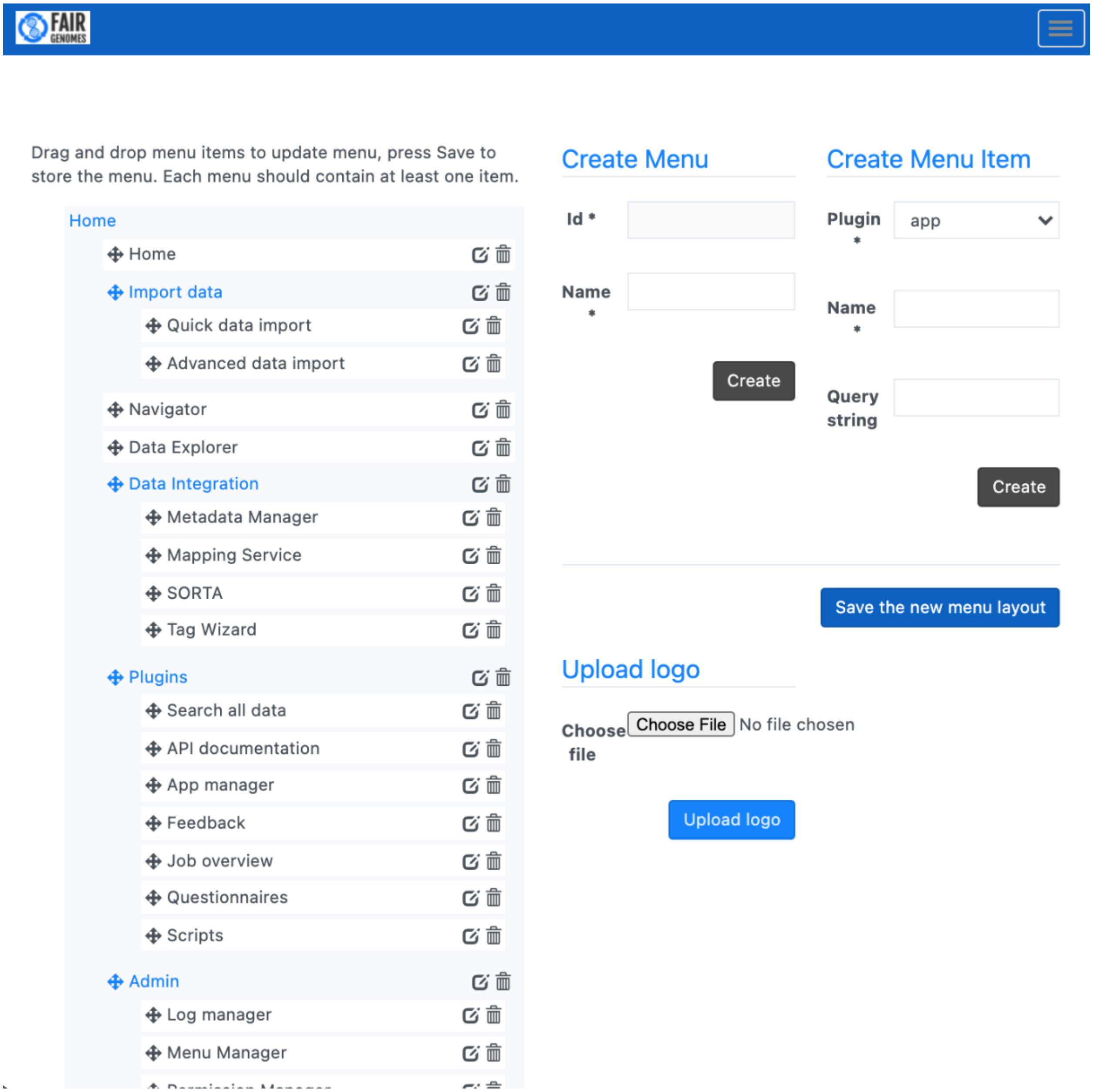

MOLGENIS also offers a detailed permission system for groups, users, packages, entities, and rows. These options are available for power users.

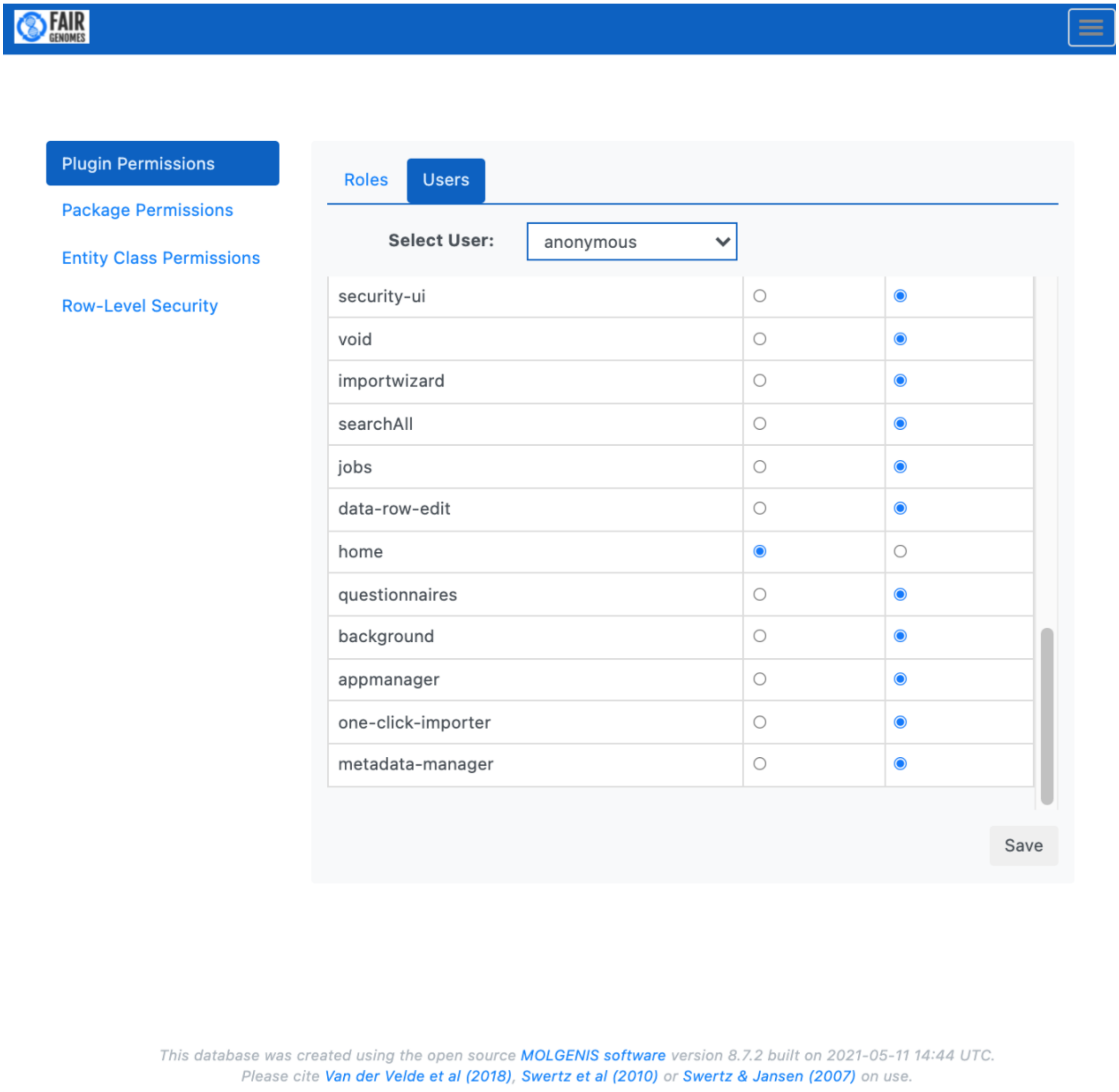

The home page can also be redesigned by clicking the Edit button on the homepage itself.

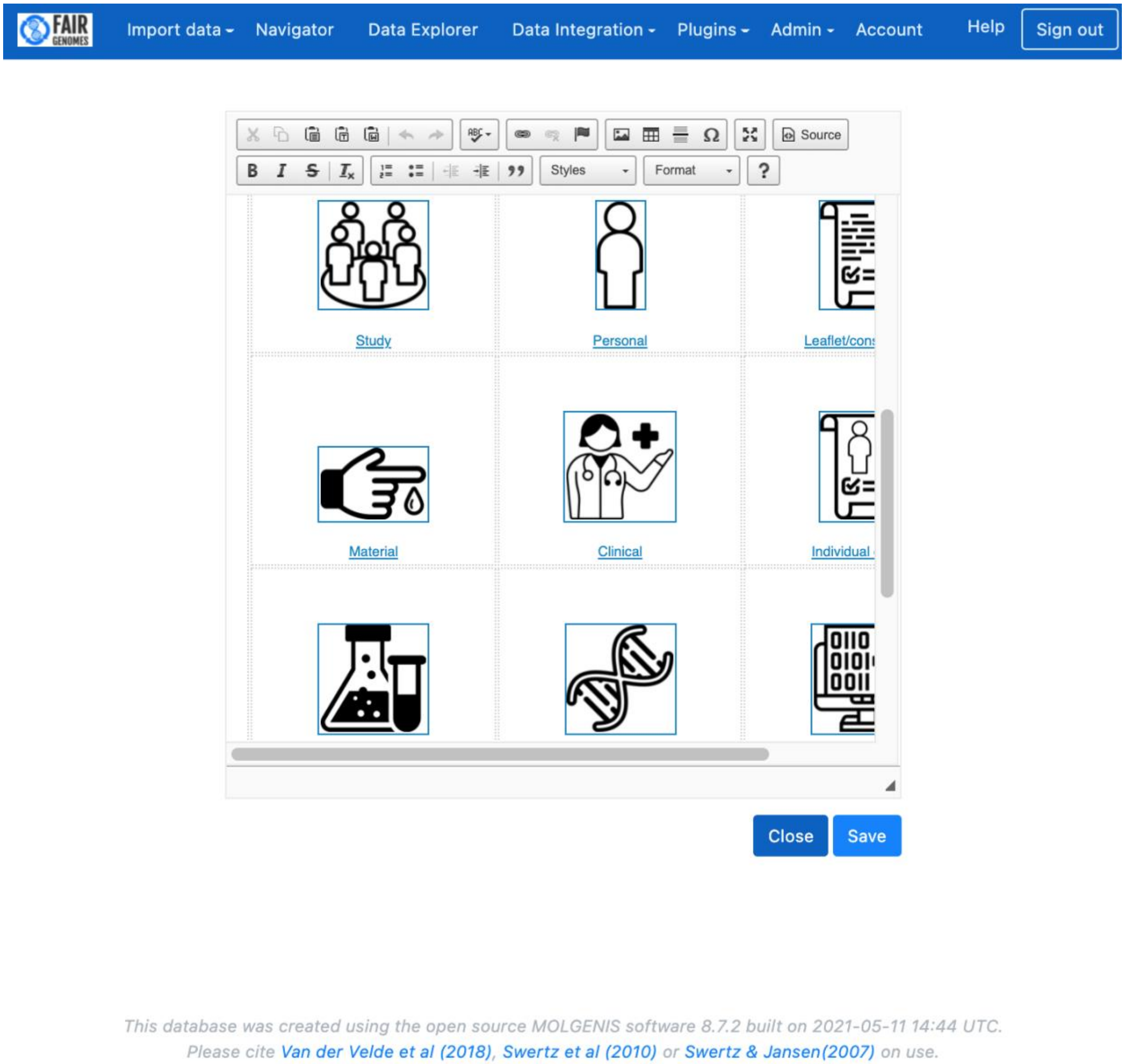

An advanced data import wizard allows uploading of bulk data using Excel or zipped TSV files.

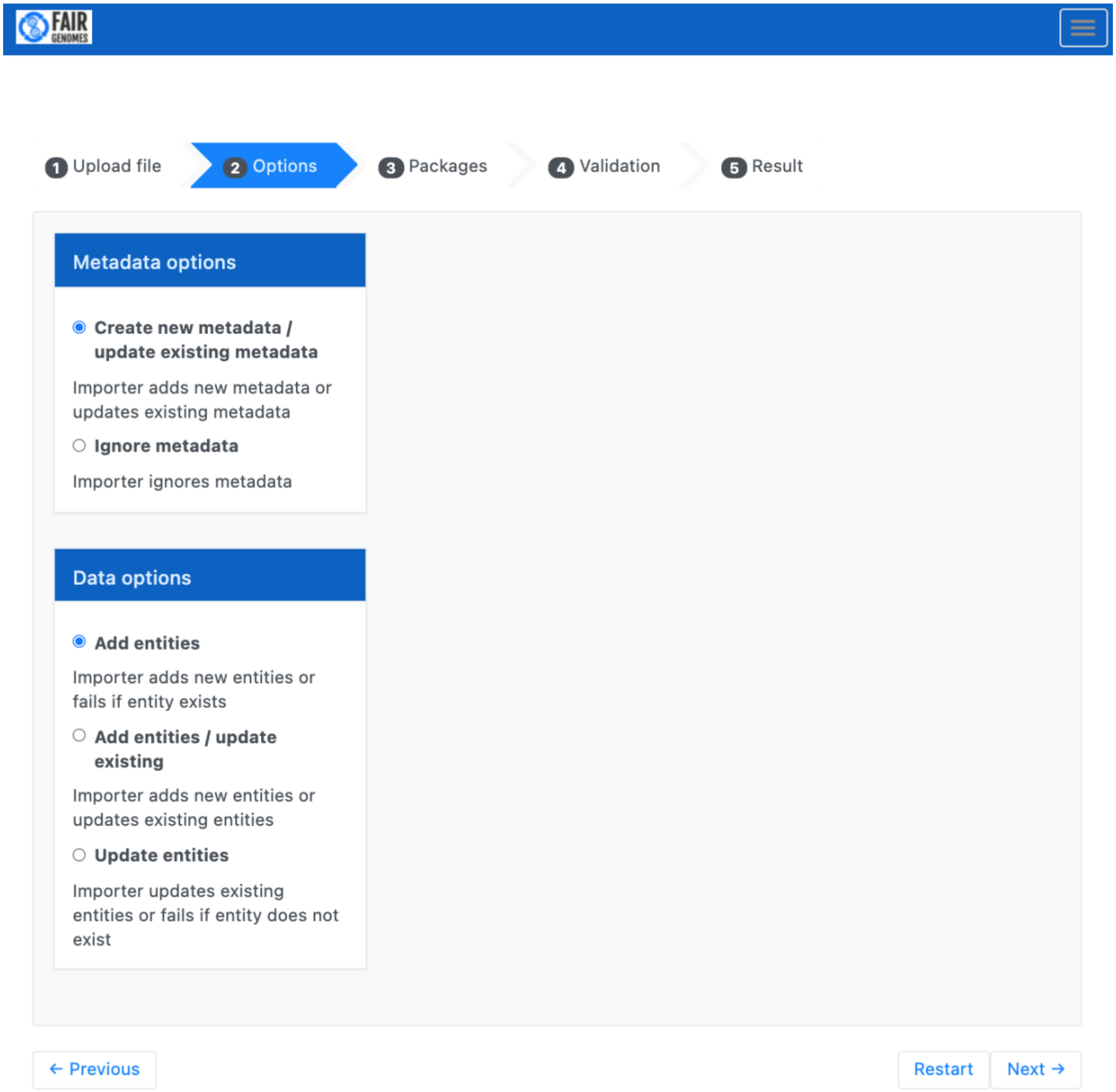

Lastly, MOLGENIS offers a variety of APIs to connect to other systems and programming environments, including:

- Data API
- Metadata API
- REST api v1
- REST api v2
- Files api
- Import api
- Permission api
- Python-api client
- R-api client
- Beacon api
- FAIR api
- RSQL operators

Example response for retrieving FAIR Genomes Sequencing data:

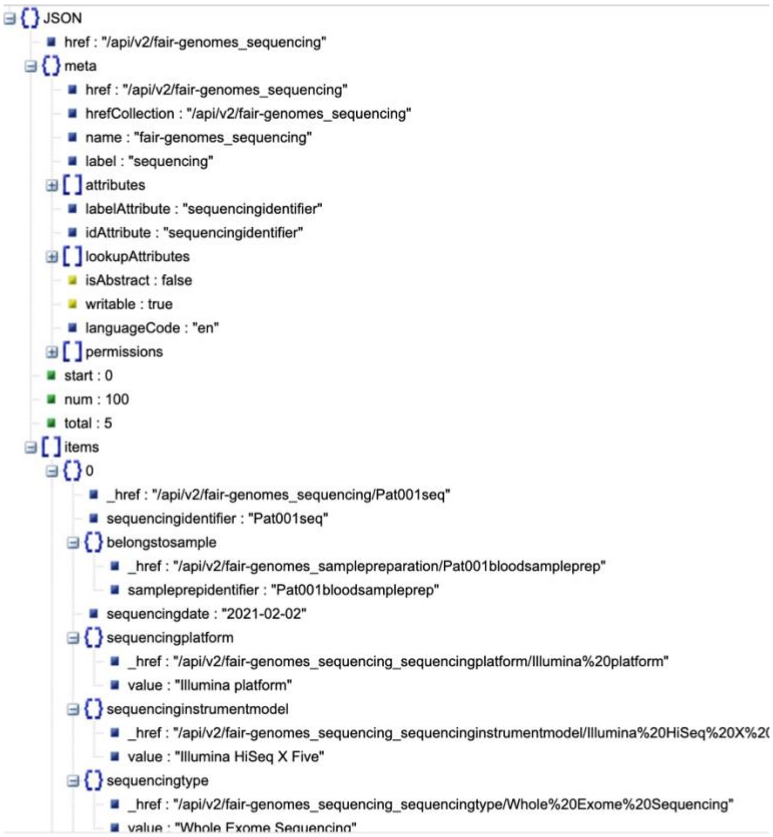

Supplement: Supplementary file 4 — Supplementary Data S4 [file 41597_2022_1265_MOESM4_ESM.pdf]
