# Supplementary material for: FAIR Genomes metadata schema promoting Next Generation Sequencing data reuse in Dutch healthcare and research
Source: Sci Data. 2022 Apr 13;9:169. doi: 10.1038/s41597-022-01265-x (PMC9008059; doi:10.1038/s41597-022-01265-x)
Supplement: Supplementary file 5 — Supplementary Data S5 [file 41597_2022_1265_MOESM5_ESM.pdf]

Supplementary Data S5:
Create FAIR Genomes EDC forms using iCRF Generator

Installation instructions

- Browse to https://github.com/aderidder/iCRFGenerator
- Click on '**Releases'**
- Download the latest iCRF Generator release for Mac or Windows
- Extract ZIP contents into a folder
- Click '**runme'** to start the application

After starting, you should see this screen:

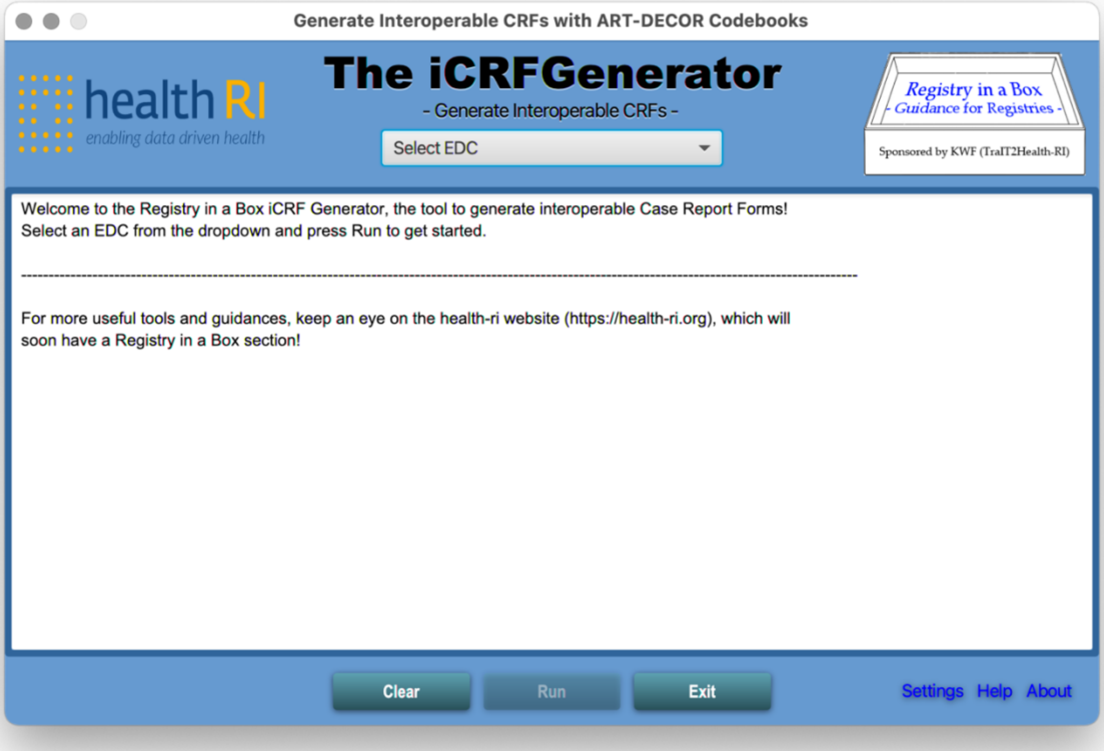

Choose your EDC and click Run.

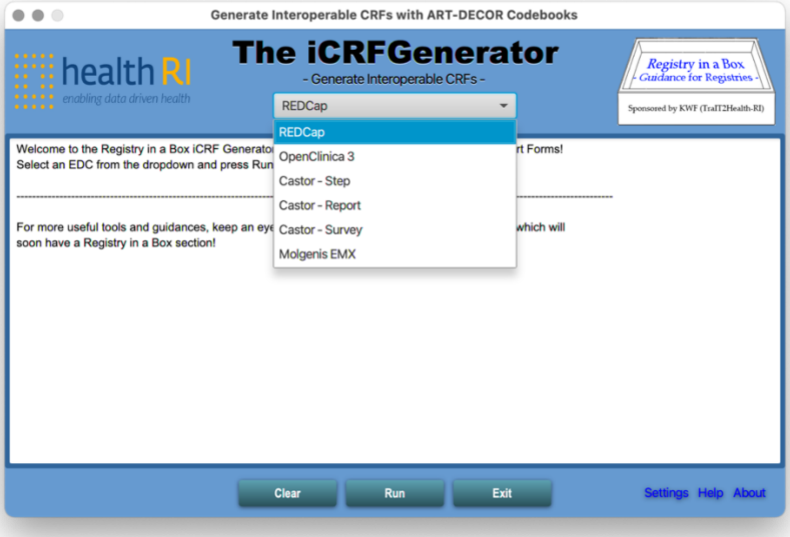

Choose the ‘FAIRGenomes’ codebook and click Next.

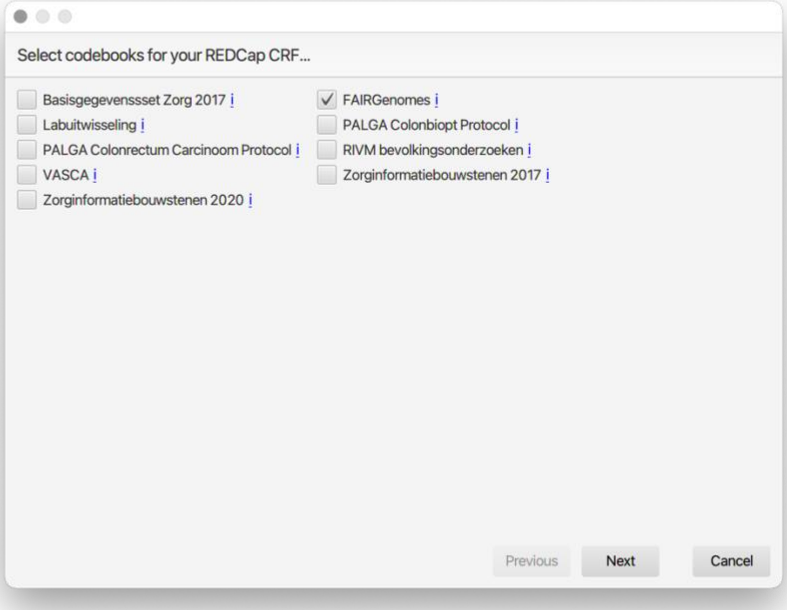

Select the appropriate codebook version, language, and click Next.

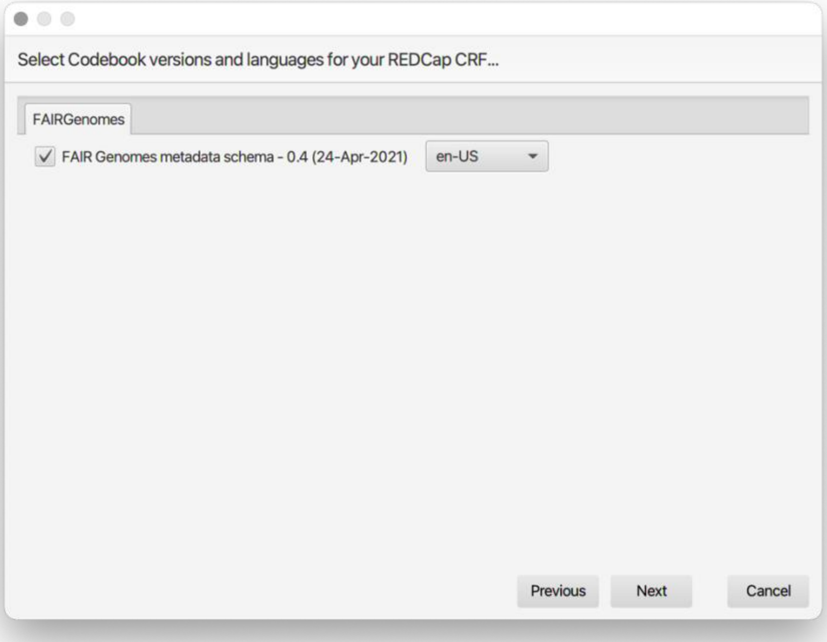

Select the items and values you wish to include in your EDC form.

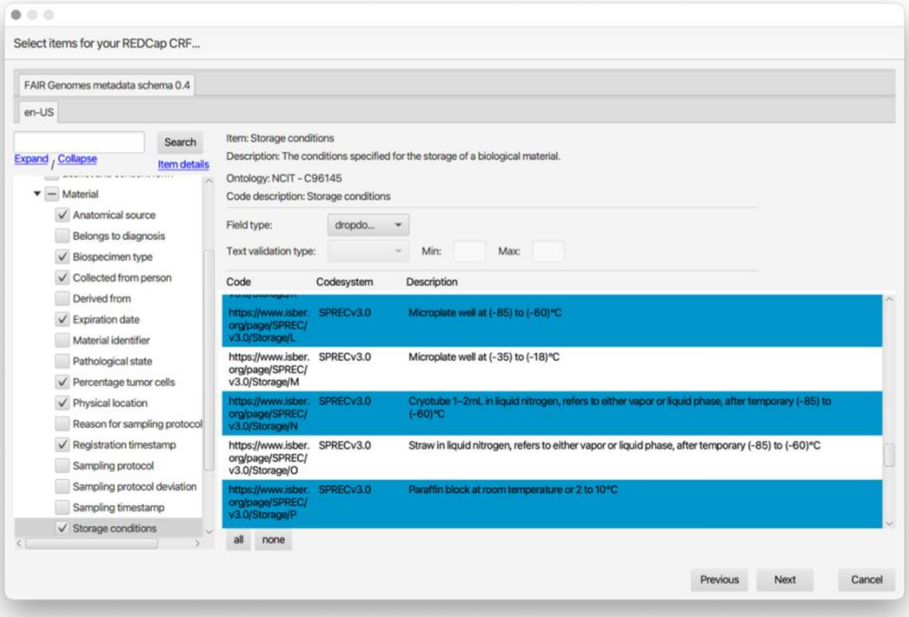

Click Next and then Finish to generate your form. This could take some time, be patient.

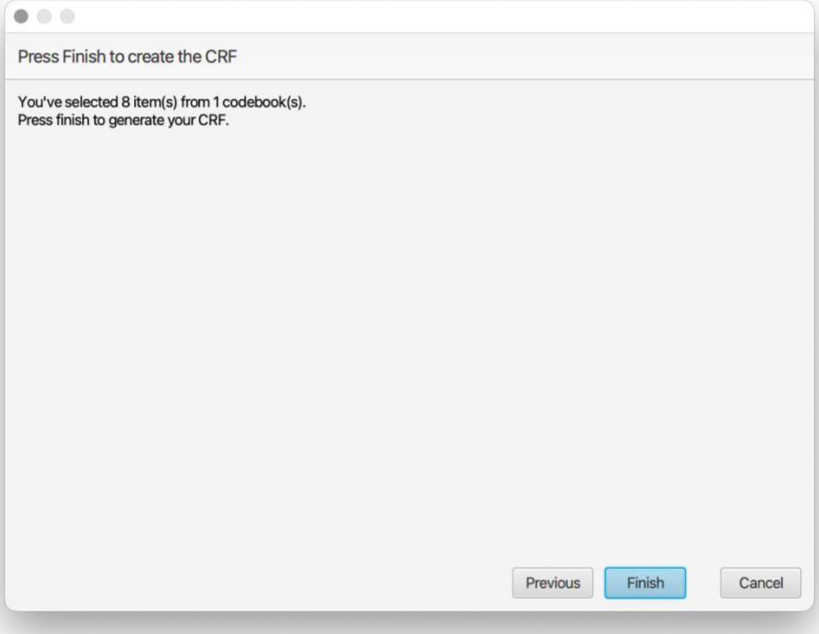

When it is ready, you may save the resulting file. The contents of the created file may look something like this and can be imported into the EDC it was intended for.

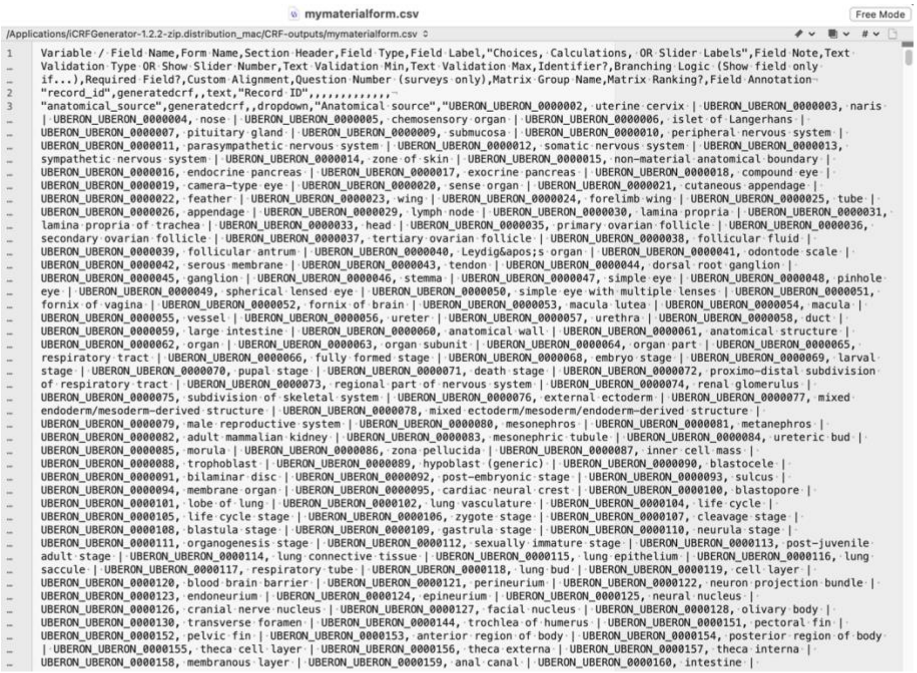


```
Variable / Field Name,Form Name,Section Header,Field Type,Field Label,"Choices, Calculations, OR Slider Labels",Field Note,Text
Validation Type OR Show Slider Number,Text Validation Min,Text Validation Max,Identifier?,Branching Logic (Show field only
if...),Required Field?,Custom Alignment,Question Number (surveys only),Matrix Group Name,Matrix Ranking?,Field Annotation
"record_id",generatedcrf,,text,"Record ID",,,,,,,,,,,,,
"anatomical_source",generatedcrf,,dropdown,"Anatomical source","UBERON_UBERON_0000002, uterine cervix | UBERON_UBERON_0000003, naris
| UBERON_UBERON_0000004, nose | UBERON_UBERON_0000005, chemosensory organ | UBERON_UBERON_0000006, islet of Langerhans |
UBERON_UBERON_0000007, pituitary gland | UBERON_UBERON_0000009, submucosa | UBERON_UBERON_0000010, peripheral nervous system |
UBERON_UBERON_0000011, parasympathetic nervous system | UBERON_UBERON_0000012, somatic nervous system | UBERON_UBERON_0000013,
sympathetic nervous system | UBERON_UBERON_0000014, zone of skin | UBERON_UBERON_0000015, non-material anatomical boundary |
UBERON_UBERON_0000016, endocrine pancreas | UBERON_UBERON_0000017, exocrine pancreas | UBERON_UBERON_0000018, compound eye |
UBERON_UBERON_0000019, camera-type eye | UBERON_UBERON_0000020, sense organ | UBERON_UBERON_0000021, cutaneous appendage |
UBERON_UBERON_0000022, feather | UBERON_UBERON_0000023, wing | UBERON_UBERON_0000024, forelimb wing | UBERON_UBERON_0000025, tube |
UBERON_UBERON_0000026, appendage | UBERON_UBERON_0000029, lymph node | UBERON_UBERON_0000030, lamina propria | UBERON_UBERON_0000031,
lamina propria of trachea | UBERON_UBERON_0000033, head | UBERON_UBERON_0000035, primary ovarian follicle | UBERON_UBERON_0000036,
secondary ovarian follicle | UBERON_UBERON_0000037, tertiary ovarian follicle | UBERON_UBERON_0000038, follicular fluid |
UBERON_UBERON_0000039, follicular antrum | UBERON_UBERON_0000040, Leydig&apos;s organ | UBERON_UBERON_0000041, odontode scale |
UBERON_UBERON_0000042, serous membrane | UBERON_UBERON_0000043, tendon | UBERON_UBERON_0000044, dorsal root ganglion |
UBERON_UBERON_0000045, ganglion | UBERON_UBERON_0000046, stemma | UBERON_UBERON_0000047, simple eye | UBERON_UBERON_0000048, pinhole
eye | UBERON_UBERON_0000049, spherical lensed eye | UBERON_UBERON_0000050, simple eye with multiple lenses | UBERON_UBERON_0000051,
fornix of vagina | UBERON_UBERON_0000052, fornix of brain | UBERON_UBERON_0000053, macula lutea | UBERON_UBERON_0000054, macula |
UBERON_UBERON_0000055, vessel | UBERON_UBERON_0000056, ureter | UBERON_UBERON_0000057, urethra | UBERON_UBERON_0000058, duct |
UBERON_UBERON_0000059, large intestine | UBERON_UBERON_0000060, anatomical wall | UBERON_UBERON_0000061, anatomical structure |
UBERON_UBERON_0000062, organ | UBERON_UBERON_0000063, organ subunit | UBERON_UBERON_0000064, organ part | UBERON_UBERON_0000065,
respiratory tract | UBERON_UBERON_0000066, fully formed stage | UBERON_UBERON_0000068, embryo stage | UBERON_UBERON_0000069, larval
stage | UBERON_UBERON_0000070, pupal stage | UBERON_UBERON_0000071, death stage | UBERON_UBERON_0000072, proximo-distal subdivision
of respiratory tract | UBERON_UBERON_0000073, regional part of nervous system | UBERON_UBERON_0000074, renal glomerulus |
UBERON_UBERON_0000075, subdivision of skeletal system | UBERON_UBERON_0000076, external ectoderm | UBERON_UBERON_0000077, mixed
endoderm/mesoderm-derived structure | UBERON_UBERON_0000078, mixed ectoderm/mesoderm/endoderm-derived structure |
UBERON_UBERON_0000079, male reproductive system | UBERON_UBERON_0000080, mesonephros | UBERON_UBERON_0000081, metanephros |
UBERON_UBERON_0000082, adult mammalian kidney | UBERON_UBERON_0000083, mesonephric tubule | UBERON_UBERON_0000084, ureteric bud |
UBERON_UBERON_0000085, morula | UBERON_UBERON_0000086, zona pellucida | UBERON_UBERON_0000087, inner cell mass |
UBERON_UBERON_0000088, trophoblast | UBERON_UBERON_0000089, hypoblast (generic) | UBERON_UBERON_0000090, blastocele |
UBERON_UBERON_0000091, bilaminar disc | UBERON_UBERON_0000092, post-embryonic stage | UBERON_UBERON_0000093, sulcus |
UBERON_UBERON_0000094, membrane organ | UBERON_UBERON_0000095, cardiac neural crest | UBERON_UBERON_0000100, blastopore |
UBERON_UBERON_0000101, lobe of lung | UBERON_UBERON_0000102, lung vasculature | UBERON_UBERON_0000104, life cycle |
UBERON_UBERON_0000105, life cycle stage | UBERON_UBERON_0000106, zygote stage | UBERON_UBERON_0000107, cleavage stage |
UBERON_UBERON_0000108, blastula stage | UBERON_UBERON_0000109, gastrula stage | UBERON_UBERON_0000110, neurula stage |
UBERON_UBERON_0000111, organogenesis stage | UBERON_UBERON_0000112, sexually immature stage | UBERON_UBERON_0000113, post-juvenile
adult stage | UBERON_UBERON_0000114, lung connective tissue | UBERON_UBERON_0000115, lung epithelium | UBERON_UBERON_0000116, lung
saccule | UBERON_UBERON_0000117, respiratory tube | UBERON_UBERON_0000118, lung bud | UBERON_UBERON_0000119, cell layer |
UBERON_UBERON_0000120, blood brain barrier | UBERON_UBERON_0000121, perineurium | UBERON_UBERON_0000122, neuron projection bundle |
UBERON_UBERON_0000123, endoneurium | UBERON_UBERON_0000124, epineurium | UBERON_UBERON_0000125, neural nucleus |
UBERON_UBERON_0000126, cranial nerve nucleus | UBERON_UBERON_0000127, facial nucleus | UBERON_UBERON_0000128, olivary body |
UBERON_UBERON_0000130, transverse foramen | UBERON_UBERON_0000144, trochlea of humerus | UBERON_UBERON_0000151, pectoral fin |
UBERON_UBERON_0000152, pelvic fin | UBERON_UBERON_0000153, anterior region of body | UBERON_UBERON_0000154, posterior region of body
| UBERON_UBERON_0000155, theca cell layer | UBERON_UBERON_0000156, theca externa | UBERON_UBERON_0000157, theca interna |
UBERON_UBERON_0000158, membranous layer | UBERON_UBERON_0000159, anal canal | UBERON_UBERON_0000160, intestine |
```
