# Supplementary material for: FAIR Genomes metadata schema promoting Next Generation Sequencing data reuse in Dutch healthcare and research
Source: Sci Data. 2022 Apr 13;9:169. doi: 10.1038/s41597-022-01265-x (PMC9008059; doi:10.1038/s41597-022-01265-x)

Supplementary Data S6:
Importing generated forms into Castor

You used iCRF Generator to create a form for Castor. Below you can see an example of a Report, which is created in XML format.

castor-report-consent.xml Free Mode

/Applications/iCRFGenerator-1.2.4-zip distrib.../CRF-outputs/castor-report-consent.xml (functions)


```xml
<?xml version="1.0" encoding="UTF-8"?><xml>
  <crf_id>65FF8881-C7F6-4AD3-9D7F-E8C7E95B2E75</crf_id>
  <optionLists>
    <og_0CFE2CA0-208E-426B-8F2A-57115B4A4B6A>
      <option_group_id>0CFE2CA0-208E-426B-8F2A-57115B4A4B6A</option_group_id>
      <option_group_name>Allow recontacting Options</option_group_name>
      <option_group_description/>
      <option_group_layout>0</option_group_layout>
      <options>
        <op_D4CA9E9F-771E-46B5-9584-2192BEFE656E>
          <option_id>D4CA9E9F-771E-46B5-9584-2192BEFE656E</option_id>
          <option_group_id>0CFE2CA0-208E-426B-8F2A-57115B4A4B6A</option_group_id>
          <option_name>Willingness to be contacted when new knowledge regarding incidental
          <option_value>http://purl.obolibrary.org/obo/ICO_0000178</option_value>
          <option_group_order>0</option_group_order>
        </op_D4CA9E9F-771E-46B5-9584-2192BEFE656E>
        <op_9D14F6D6-2491-40B0-8806-856FC4C33992>
          <option_id>9D14F6D6-2491-40B0-8806-856FC4C33992</option_id>
          <option_group_id>0CFE2CA0-208E-426B-8F2A-57115B4A4B6A</option_group_id>
          <option_name>Willingness to be contacted when new knowledge regarding the diagno
          <option_value>http://purl.obolibrary.org/obo/NCIT_C15220</option_value>
          <option_group_order>1</option_group_order>
        </op_9D14F6D6-2491-40B0-8806-856FC4C33992>
        <op_7947CF26-C98A-4BAF-8BB9-563DAC67FE42>
          <option_id>7947CF26-C98A-4BAF-8BB9-563DAC67FE42</option_id>
          <option_group_id>0CFE2CA0-208E-426B-8F2A-57115B4A4B6A</option_group_id>
          <option_name>Willingness to be contacted to collect additional material or data
          <option_value>http://purl.obolibrary.org/obo/NCIT_C25662</option_value>
          <option_group_order>2</option_group_order>
        </op_7947CF26-C98A-4BAF-8BB9-563DAC67FE42>
      </options>
    </og_0CFE2CA0-208E-426B-8F2A-57115B4A4B6A>
    <og_AF9F846C-3FC9-45EE-B2C0-E23186CE0EBF>
      <option_group_id>AF9F846C-3FC9-45EE-B2C0-E23186CE0EBF</option_group_id>
      <option_group_name>Collected by Options</option_group_name>
      <option_group_description/>
      <option_group_layout>0</option_group_layout>
      <options>
        <op_34F1505B-751E-48F5-8B4C-E1C9884D6297>
          <option_id>34F1505B-751E-48F5-8B4C-E1C9884D6297</option_id>
          <option_group_id>AF9F846C-3FC9-45EE-B2C0-E23186CE0EBF</option_group_id>
          <option_name>Information was sought but not found (e.g., patient was asked but d
          <option_value>http://terminology.hl7.org/CodeSystem/v3-NullFlavor#ASKU</option_v
          <option_group_order>0</option_group_order>
        </op_34F1505B-751E-48F5-8B4C-E1C9884D6297>
        <op_D2C1A620-46E5-4C27-BF82-9AD23E5071FF>
```


L: 95 C: 29 XML Saved: 7/2/21, 8:54:59 AM 911,156 / 70,702 / 12,850 4 100%

First, within Castor, create a study by filling in the Create New Study form.

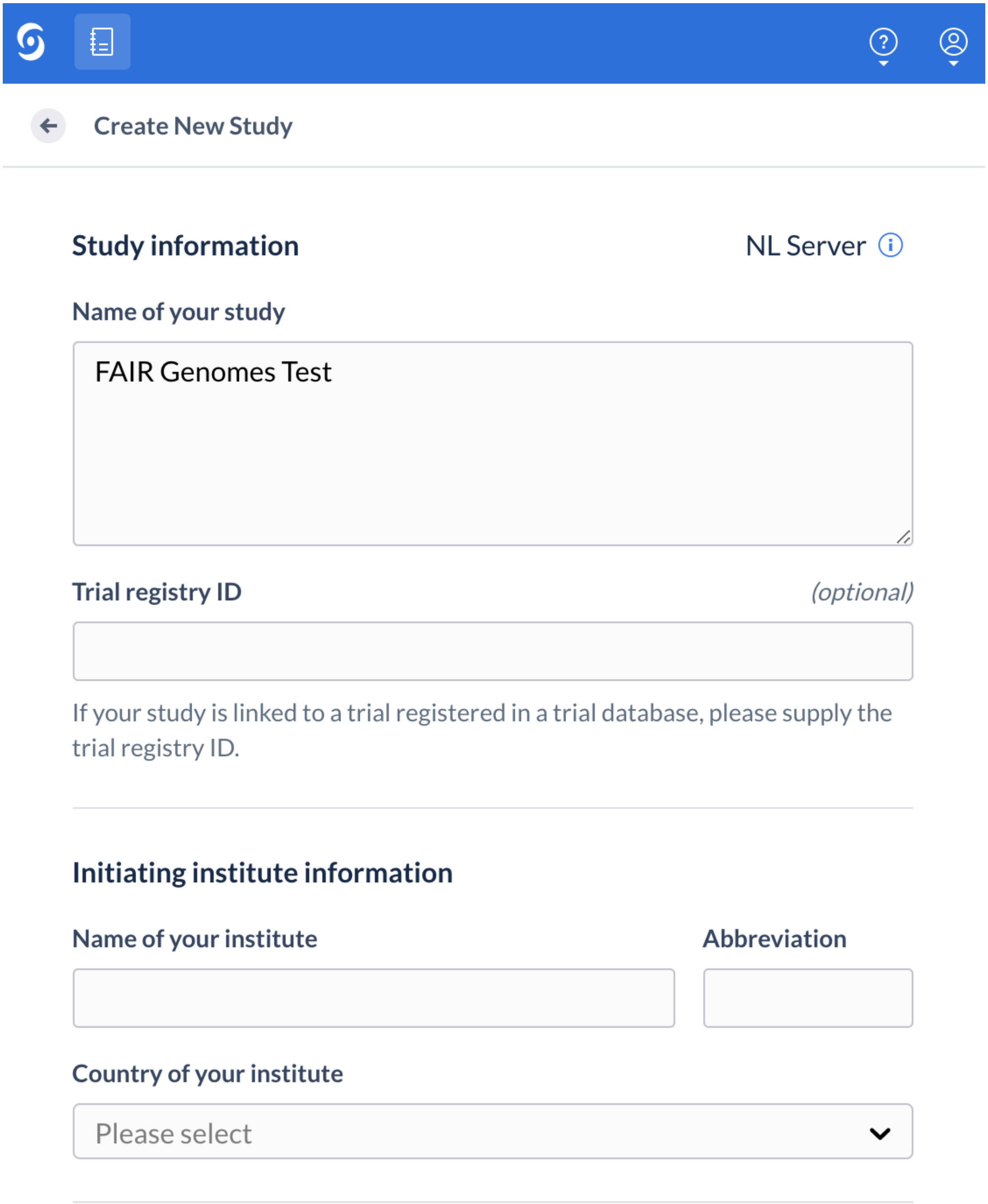

Within this study, you can add Reports, Surveys and Steps, which can be created by iCRF Generator. In this example, we will import a Report. Go to Structure → Reports.

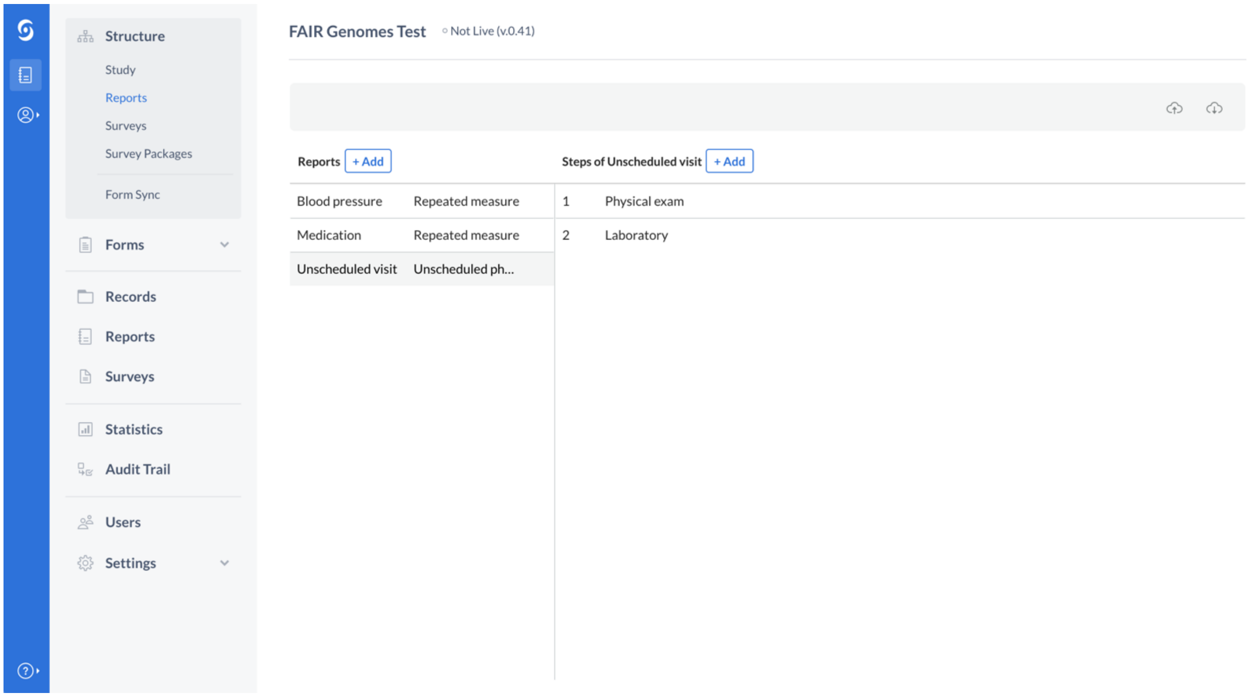

Click on Import structure, select the appropriate file, and click Upload.

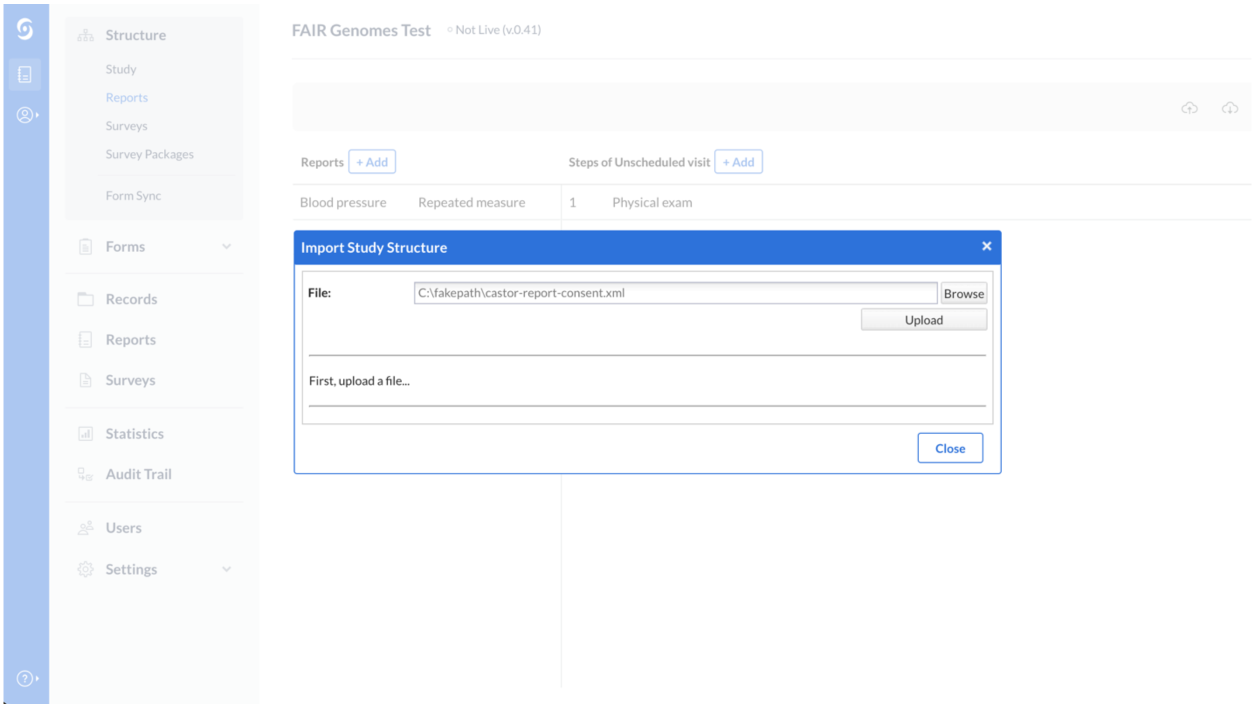

The report should now have been added. It can be seen by clicking Open in form editor.

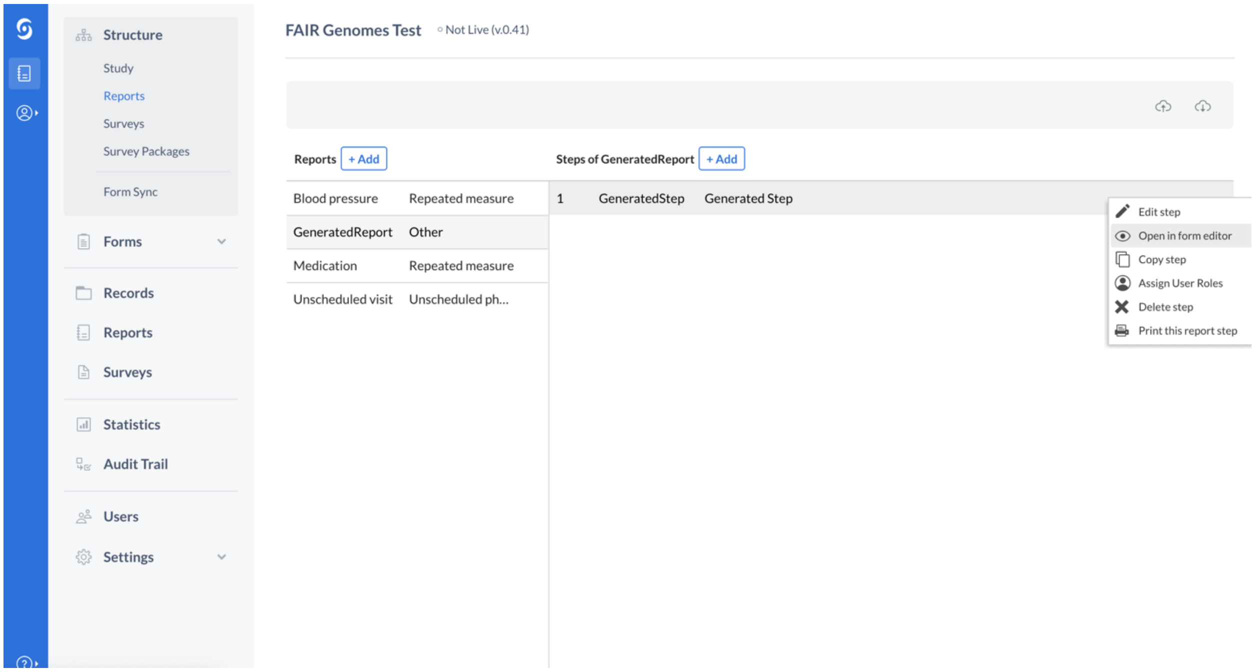

Here, you can finalize the report if necessary and start to use it as part of your study.

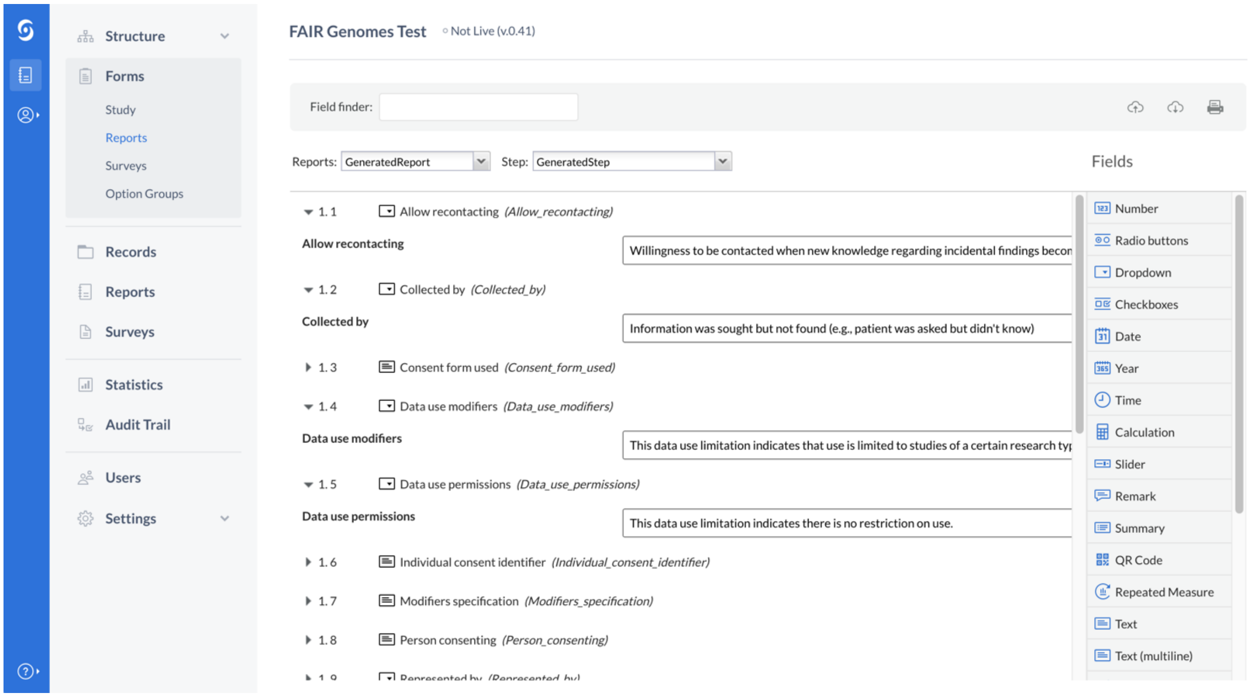

Supplement: Supplementary file 6 — Supplementary Data S6 [file 41597_2022_1265_MOESM6_ESM.pdf]
