# Supplementary material for: FAIR Genomes metadata schema promoting Next Generation Sequencing data reuse in Dutch healthcare and research
Source: Sci Data. 2022 Apr 13;9:169. doi: 10.1038/s41597-022-01265-x (PMC9008059; doi:10.1038/s41597-022-01265-x)

Supplementary Data S7:
Importing generated forms into REDCap

You used iCRF Generator to create a REDCap form. Below you can see an example of a form, which is created in CSV format.

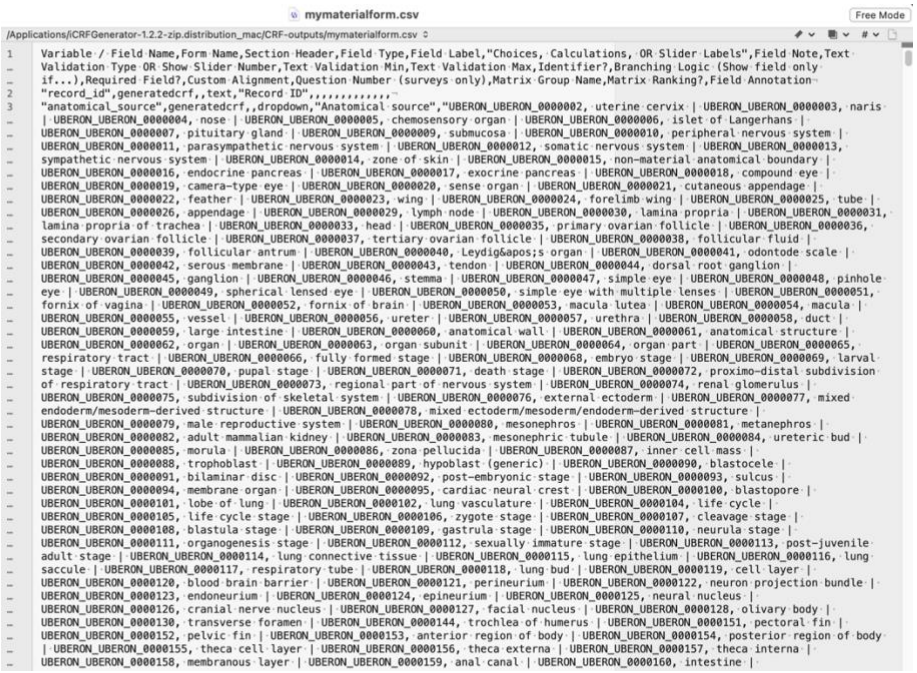
mymaterialform.csv

Free Mode

/Applications/iCRFGenerator-1.2.2-zip.distribution_mac/CRF-outputs/mymaterialform.csv

```
Variable / Field Name,Form Name,Section Header,Field Type,Field Label,"Choices, Calculations, OR Slider Labels",Field Note,Text Validation Type OR Show Slider Number,Text Validation Min,Text Validation Max,Identifier?,Branching Logic (Show field only if...),Required Field?,Custom Alignment,Question Number (surveys only),Matrix Group Name,Matrix Ranking?,Field Annotation
"record_id",generatedcrf,,text,"Record ID",,,,,,,,,,,,,
"anatomical_source",generatedcrf,,dropdown,"Anatomical source","UBERON_UBERON_0000002, uterine cervix | UBERON_UBERON_0000003, naris | UBERON_UBERON_0000004, nose | UBERON_UBERON_0000005, chemosensory organ | UBERON_UBERON_0000006, islet of Langerhans | UBERON_UBERON_0000007, pituitary gland | UBERON_UBERON_0000009, submucosa | UBERON_UBERON_0000010, peripheral nervous system | UBERON_UBERON_0000011, parasympathetic nervous system | UBERON_UBERON_0000012, somatic nervous system | UBERON_UBERON_0000013, sympathetic nervous system | UBERON_UBERON_0000014, zone of skin | UBERON_UBERON_0000015, non-material anatomical boundary | UBERON_UBERON_0000016, endocrine pancreas | UBERON_UBERON_0000017, exocrine pancreas | UBERON_UBERON_0000018, compound eye | UBERON_UBERON_0000019, camera-type eye | UBERON_UBERON_0000020, sense organ | UBERON_UBERON_0000021, cutaneous appendage | UBERON_UBERON_0000022, feather | UBERON_UBERON_0000023, wing | UBERON_UBERON_0000024, forelimb wing | UBERON_UBERON_0000025, tube | UBERON_UBERON_0000026, appendage | UBERON_UBERON_0000029, lymph node | UBERON_UBERON_0000030, lamina propria | UBERON_UBERON_0000031, lamina propria of trachea | UBERON_UBERON_0000033, head | UBERON_UBERON_0000035, primary ovarian follicle | UBERON_UBERON_0000036, secondary ovarian follicle | UBERON_UBERON_0000037, tertiary ovarian follicle | UBERON_UBERON_0000038, follicular fluid | UBERON_UBERON_0000039, follicular antrum | UBERON_UBERON_0000040, Leydig&apos;s organ | UBERON_UBERON_0000041, odontode scale | UBERON_UBERON_0000042, serous membrane | UBERON_UBERON_0000043, tendon | UBERON_UBERON_0000044, dorsal root ganglion | UBERON_UBERON_0000045, ganglion | UBERON_UBERON_0000046, stemma | UBERON_UBERON_0000047, simple eye | UBERON_UBERON_0000048, pinhole eye | UBERON_UBERON_0000049, spherical lensed eye | UBERON_UBERON_0000050, simple eye with multiple lenses | UBERON_UBERON_0000051, fornix of vagina | UBERON_UBERON_0000052, fornix of brain | UBERON_UBERON_0000053, macula lutea | UBERON_UBERON_0000054, macula | UBERON_UBERON_0000055, vessel | UBERON_UBERON_0000056, ureter | UBERON_UBERON_0000057, urethra | UBERON_UBERON_0000058, duct | UBERON_UBERON_0000059, large intestine | UBERON_UBERON_0000060, anatomical wall | UBERON_UBERON_0000061, anatomical structure | UBERON_UBERON_0000062, organ | UBERON_UBERON_0000063, organ subunit | UBERON_UBERON_0000064, organ part | UBERON_UBERON_0000065, respiratory tract | UBERON_UBERON_0000066, fully formed stage | UBERON_UBERON_0000068, embryo stage | UBERON_UBERON_0000069, larval stage | UBERON_UBERON_0000070, pupal stage | UBERON_UBERON_0000071, death stage | UBERON_UBERON_0000072, proximo-distal subdivision of respiratory tract | UBERON_UBERON_0000073, regional part of nervous system | UBERON_UBERON_0000074, renal glomerulus | UBERON_UBERON_0000075, subdivision of skeletal system | UBERON_UBERON_0000076, external ectoderm | UBERON_UBERON_0000077, mixed endoderm/mesoderm-derived structure | UBERON_UBERON_0000078, mixed ectoderm/mesoderm/endoderm-derived structure | UBERON_UBERON_0000079, male reproductive system | UBERON_UBERON_0000080, mesonephros | UBERON_UBERON_0000081, metanephros | UBERON_UBERON_0000082, adult mammalian kidney | UBERON_UBERON_0000083, mesonephric tubule | UBERON_UBERON_0000084, ureteric bud | UBERON_UBERON_0000085, morula | UBERON_UBERON_0000086, zona pellucida | UBERON_UBERON_0000087, inner cell mass | UBERON_UBERON_0000088, trophoblast | UBERON_UBERON_0000089, hypoblast (generic) | UBERON_UBERON_0000090, blastocele | UBERON_UBERON_0000091, bilaminar disc | UBERON_UBERON_0000092, post-embryonic stage | UBERON_UBERON_0000093, sulcus | UBERON_UBERON_0000094, membrane organ | UBERON_UBERON_0000095, cardiac neural crest | UBERON_UBERON_0000100, blastopore | UBERON_UBERON_0000101, lobe of lung | UBERON_UBERON_0000102, lung vasculature | UBERON_UBERON_0000104, life cycle | UBERON_UBERON_0000105, life cycle stage | UBERON_UBERON_0000106, zygote stage | UBERON_UBERON_0000107, cleavage stage | UBERON_UBERON_0000108, blastula stage | UBERON_UBERON_0000109, gastrula stage | UBERON_UBERON_0000110, neurula stage | UBERON_UBERON_0000111, organogenesis stage | UBERON_UBERON_0000112, sexually immature stage | UBERON_UBERON_0000113, post-juvenile adult stage | UBERON_UBERON_0000114, lung connective tissue | UBERON_UBERON_0000115, lung epithelium | UBERON_UBERON_0000116, lung saccule | UBERON_UBERON_0000117, respiratory tube | UBERON_UBERON_0000118, lung bud | UBERON_UBERON_0000119, cell layer | UBERON_UBERON_0000120, blood brain barrier | UBERON_UBERON_0000121, perineurium | UBERON_UBERON_0000122, neuron projection bundle | UBERON_UBERON_0000123, endoneurium | UBERON_UBERON_0000124, epineurium | UBERON_UBERON_0000125, neural nucleus | UBERON_UBERON_0000126, cranial nerve nucleus | UBERON_UBERON_0000127, facial nucleus | UBERON_UBERON_0000128, olivary body | UBERON_UBERON_0000130, transverse foramen | UBERON_UBERON_0000144, trochlea of humerus | UBERON_UBERON_0000151, pectoral fin | UBERON_UBERON_0000152, pelvic fin | UBERON_UBERON_0000153, anterior region of body | UBERON_UBERON_0000154, posterior region of body | UBERON_UBERON_0000155, theca cell layer | UBERON_UBERON_0000156, theca externa | UBERON_UBERON_0000157, theca interna | UBERON_UBERON_0000158, membranous layer | UBERON_UBERON_0000159, anal canal | UBERON_UBERON_0000160, intestine |
```

To import the form, launch REDCap and go to the Data Dictionary menu.

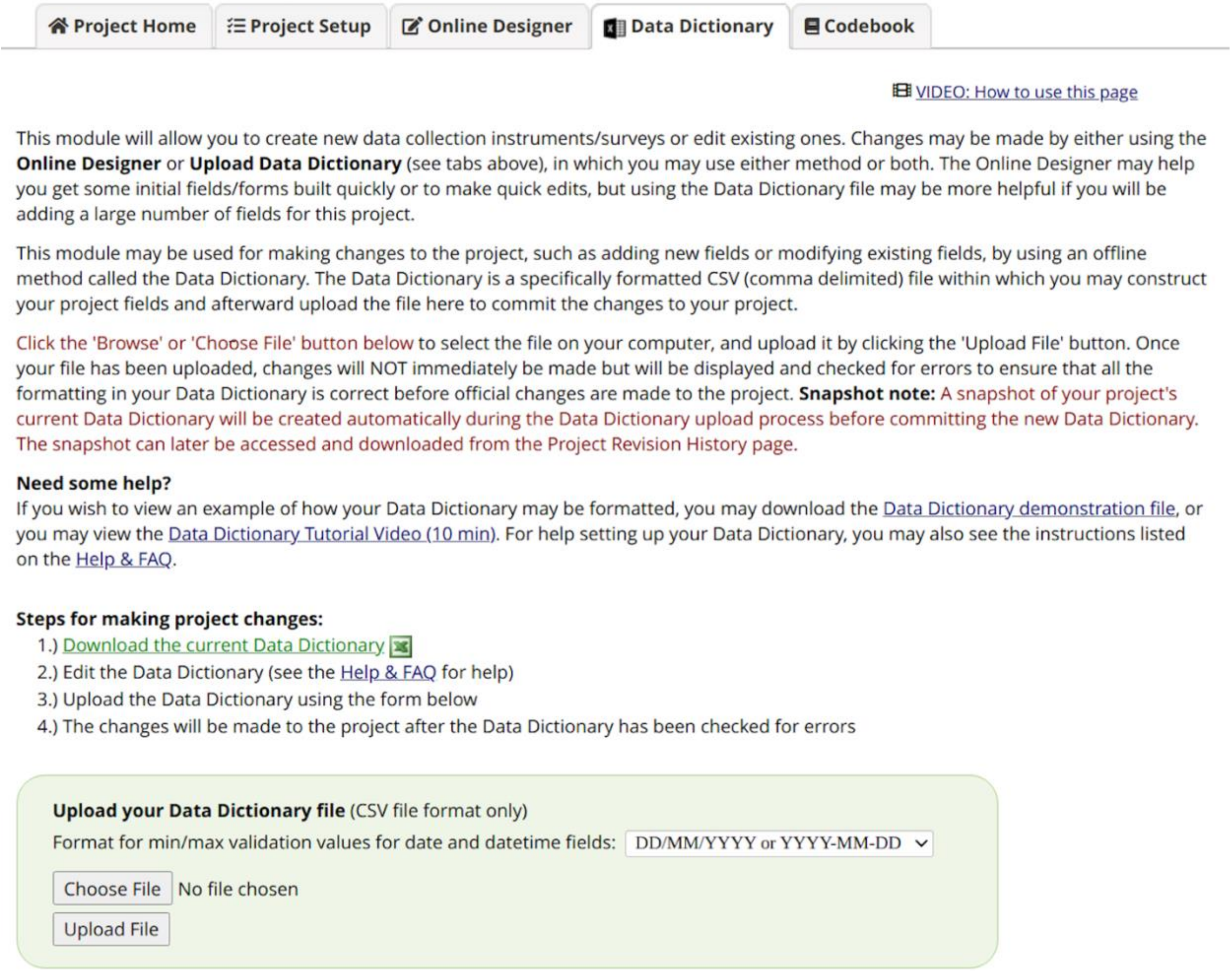

Here, select the appropriate file and click Commit Changes.

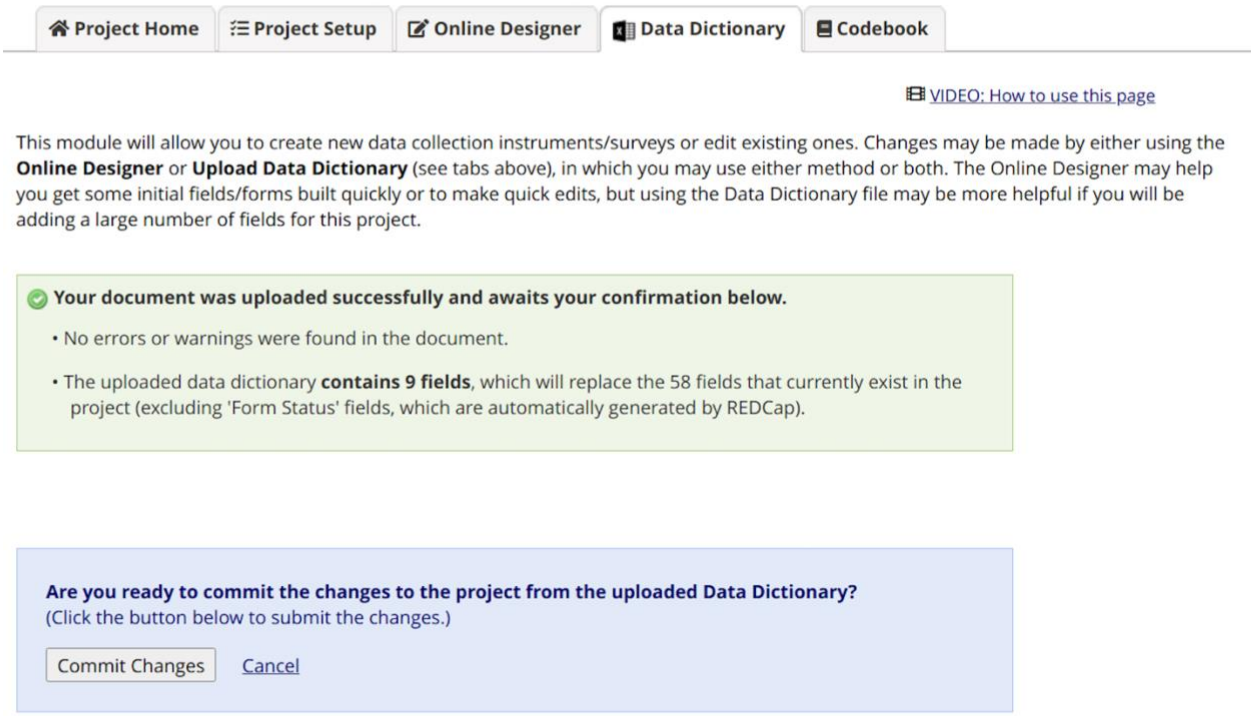

This should be followed by a success message.

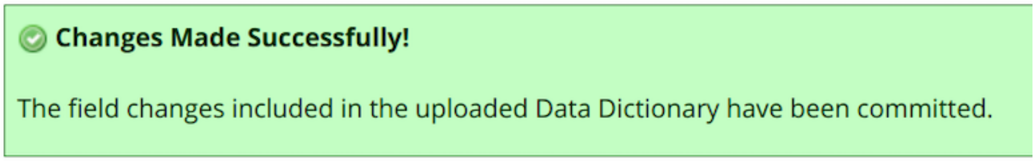

The form can be edited in the Online Designer menu, if adjustments are required.

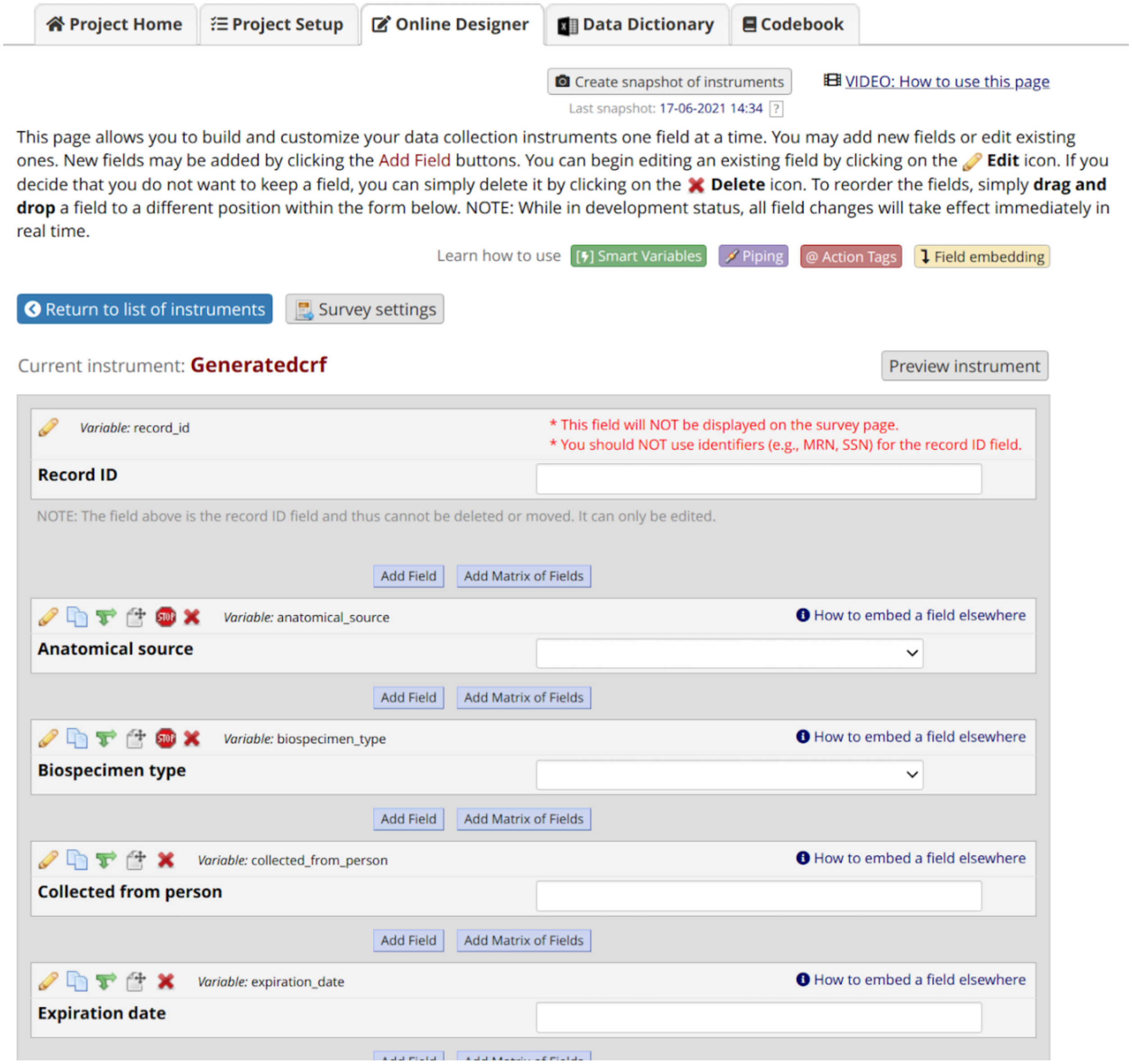

By clicking Preview instrument, a preview can be seen of what this form will look like when presented to a user.

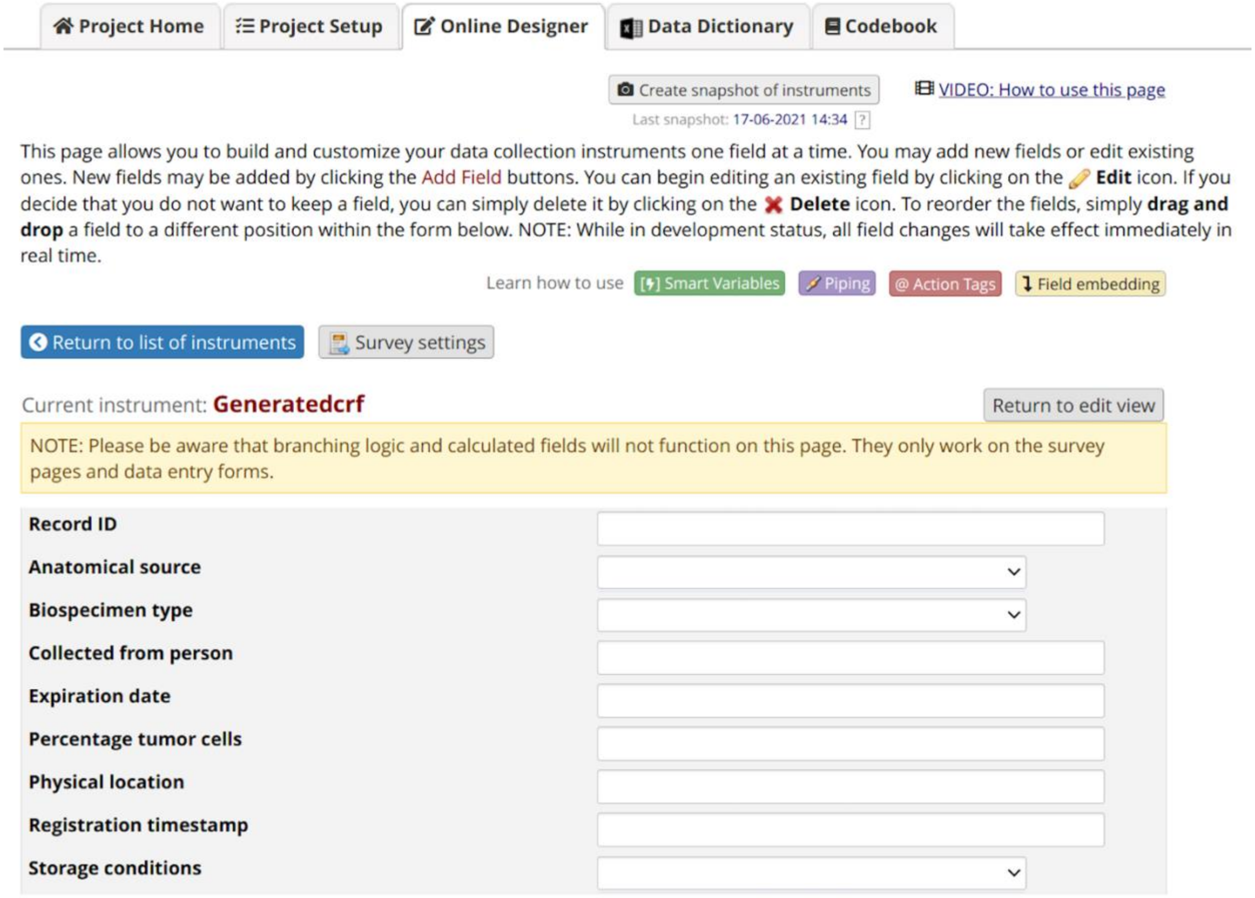

Supplement: Supplementary file 7 — Supplementary Data S7 [file 41597_2022_1265_MOESM7_ESM.pdf]
